# The *Arabidopsis* SWI/SNF protein BAF60 mediates seedling growth control by modulating DNA accessibility

**DOI:** 10.1186/s13059-017-1246-7

**Published:** 2017-06-15

**Authors:** Teddy Jégu, Alaguraj Veluchamy, Juan S. Ramirez-Prado, Charley Rizzi-Paillet, Magalie Perez, Anaïs Lhomme, David Latrasse, Emeline Coleno, Serge Vicaire, Stéphanie Legras, Bernard Jost, Martin Rougée, Fredy Barneche, Catherine Bergounioux, Martin Crespi, Magdy M. Mahfouz, Heribert Hirt, Cécile Raynaud, Moussa Benhamed

**Affiliations:** 1 Institut of Plant Sciences Paris-Saclay (IPS2), UMR 9213/UMR1403, CNRS, INRA, Université Paris-Sud, Université d’Evry, Université Paris-Diderot, Sorbonne Paris-Cité, Bâtiment 630, 91405 Orsay, France; 2 Division of Biological and Environmental Sciences and Engineering, King Abdullah University of Science and Technology, Thuwal, 23955-6900 Kingdom of Saudi Arabia; 3 Plateforme Biopuces et séquençage, IGBMC, 1 rue Laurent Fries Parc d’Innovation, 67400 Illkirch, France; 4 Ecole Normale Supérieure, PSL Research University, Institut de Biologie de l’Ecole Normale Supérieure (IBENS), CNRS UMR 8197, INSERM U1024, 46 rue d’Ulm, F-75005 Paris, France; 5 Present address: Howard Hughes Medical Institute, Department of Molecular Biology, Massachusetts General Hospital, Boston, MA 02114 USA; 6 Present address: Department of Genetics, Harvard Medical School, Boston, MA 02114 USA

**Keywords:** SWI/SNF, Chromatin, Morphogenesis, Phytochrome Interacting Factor, G-box, DNA accessibility, PIF4

## Abstract

**Background:**

Plant adaptive responses to changing environments involve complex molecular interplays between intrinsic and external signals. Whilst much is known on the signaling components mediating diurnal, light, and temperature controls on plant development, their influence on chromatin-based transcriptional controls remains poorly explored.

**Results:**

In this study we show that a SWI/SNF chromatin remodeler subunit, BAF60, represses seedling growth by modulating DNA accessibility of hypocotyl cell size regulatory genes. BAF60 binds nucleosome-free regions of multiple G box-containing genes, opposing in *cis* the promoting effect of the photomorphogenic and thermomorphogenic regulator Phytochrome Interacting Factor 4 (PIF4) on hypocotyl elongation. Furthermore, *BAF60* expression level is regulated in response to light and daily rhythms.

**Conclusions:**

These results unveil a short path between a chromatin remodeler and a signaling component to fine-tune plant morphogenesis in response to environmental conditions.

**Electronic supplementary material:**

The online version of this article (doi:10.1186/s13059-017-1246-7) contains supplementary material, which is available to authorized users.

## Background

Plants take advantage of a complex series of sensory receptors to perceive and respond to environmental cues such as the intensity, wavelength, direction, and duration of ambient light signals. For example, the Pr/Pfr reversible forms of red (R) and far-red (FR) sensing phytochrome photoreceptors allow plants to discriminate between mid-day and dusk periods, or shade and plain light conditions, notably because of rapid changes in the R/FR ratio. UV-A/blue light cryptochrome photoreceptors also contribute to control plant adaptive responses such as photomorphogenesis, the developmental transition that seedlings undergo on their first exposure to light. Light signal transduction involves multiple central integrators and sequence-specific effectors [1, 2]. Among them, a subset of the basic helix-loop-helix (bHLH) transcription factors called phytochrome-interacting factors (PIFs) physically interact with both phytochrome and cryptochrome photoreceptors under different light conditions [3, 4].

PIF proteins (PIF1, PIF3, PIF4, PIF5, PIF6, and PIF7 in *Arabidopsis*) accumulate in the dark to promote etiolated development (skotomorphogenesis). Distinct PIF family members promote hypocotyl elongation by inducing the expression of cell size regulatory genes, notably via the modulation of auxin accumulation in cotyledons [5]. Accordingly, *pifq* mutant plants, lacking a functional *PIF1*, *PIF3*, *PIF4*, and *PIF5* quartet, display a short hypocotyl phenotype [6]. Expression, stability, and activity of the PIFs are also tightly controlled by light and temperature during seedling and adult developmental stages via a combination of transcriptional and post-translational controls [6–9]. Among them, PIF4 represents a central hub for modulating various facets of plant morphogenesis, through the integration of multiple environmental signals, such as photomorphogenesis, the shade avoidance response (SAR), and thermomorphogenesis [10–12]. The expression and activity of PIF4 are also regulated by the circadian clock, possibly influencing time-dependent transcriptional responses to environmental cues [13].

The bZIP ELONGATED HYPOCOTYL 5 (HY5) transcription factor is another major regulator of thermo- and photomorphogenesis. *HY5* regulates hypocotyl elongation in response to light conditions [14], suggesting that it acts downstream of phytochrome A (phyA), phyB, cryptochrome, and UVR8 photoreceptors [14–16]. The protein stability of PIF4 and HY5 is also regulated by the COP1-DET1 signal integrators acting downstream of these photoreceptors [17, 18]. They control a wide repertoire of target genes, largely by binding to consensus sequences such as G-boxes [14, 19]. In fact, it was recently proved that HY5 inhibits PIF4-directed hypocotyl elongation by competitive chromatin binding to common targets. Such result reveals two independent pathways of PIF4 regulation, one involving DET1/COP1 and other HY5 [20].

In addition to such sequence-specific factors, accumulating evidence indicates that chromatin modifications, which are crucial components of transcription regulation, participate in light-mediated gene expression [21, 22]. Since the first piece of evidence that increased acetylation of histone H3 and H4 of the *Pisum sativum PetE* gene promoter correlates with light-induced transcription [23], several functional studies have shown the influence of histone acetyltransferase and deacetylase activities that oppositely balance histone acetylation levels on *Arabidopsis* photomorphogenesis [24, 25]. Interestingly, in darkness the histone deacetylase HDA15 can associate with PIF3 and repress expression of photomorphogenic genes, providing a molecular link between a chromatin-modifying activity and light-responsive elements. Accordingly, PIF3 does not contribute to the HDA15 influence on hypocotyl elongation upon light exposure [26]. Profiling of light-induced chromatin state changes along the *Arabidopsis* genome has further unraveled the wide extent of a chromatin-based program associated with light perception and photomorphogenesis [27–30], but the molecular links triggering light- and sequence-specific chromatin dynamics remain poorly understood.

Chromatin-based transcriptional regulation relies on the deposition and removal of multiple histone and DNA modifications, but also on ATP-dependent chromatin remodeling complexes (CRCs) that modulate histone–DNA contacts [31]. These multi-protein complexes regulate access of genomic regions to transcription factors and to the transcriptional machinery by influencing the structure, dynamic incorporation/eviction of histone variants, or nucleosome positioning. A distinctive feature of CRCs is the presence of a central ATPase domain belonging to the SWI2/SNF2 family [32, 33]. In *Arabidopsis*, PICKLE, a member of the ATPases CHD3 subfamily (also referred to as PKL or GYMNOS/Suppressor of *slr2* [SSL2]/CYTOKININ-HYPERSENSITIVE2) [34] regulates multiple plant developmental processes, including embryonic development, seed germination, root meristem activity, and photomorphogenesis [35–39]. PKL was found to physically interact with HY5 and to influence hypocotyl elongation by triggering an increase of histone H3 lysine 27 trimethylation (H3K27me3) of several cell elongation-related genes in response to light [39], a chromatin signature of *Polycomb*-repressed transcriptional states [40]. However, how the respective activities of chromatin remodeling and light signaling integrate and contribute to control plant adaptive responses remains to be explored on a genome-wide scale.

Based on sequence similarity with metazoan DNA sequences, the *Arabidopsis* genome encompasses multiple CRCs [41]. These include four SWI2/SNF2 ATPases (BRM, SPLAYED [SYD], MINU1/CHR12, and MINU2/CHR23), four SWI3 proteins (SWI3A to SWI3D), two ACTIN RELATED PROTEINS predicted to belong to SWI/SNF complexes (ARP4 and ARP7), a single protein termed BUSHY (BSH), and two SWI/SNF ASSOCIATED PROTEINS 73 (SWP73A/CHC2 and SWP73B/CHC1), also called BAF60 [42–47]. In addition to their primary activity modulating histone–DNA interactions, and the accessibility of sequence-specific binding proteins onto DNA [48], CRCs also impinge on various other chromatin-related processes such as histone acetylation, methylation, phosphorylation, and ubiquitination, altogether influencing local chromatin status [49]. These complexes may also impact on the formation of local chromatin loops to modulate gene expression [47, 50–52]. Here, we report that the SWI/SNF chromatin-remodeling factor BAF60 negatively regulates hypocotyl elongation under light and high temperature conditions, and locally represses the expression of multiple cell size regulatory genes by influencing DNA accessibility. Hundreds of BAF60 target genes are shared with PIF4, raising the possibility that BAF60-containing CRCs represents a key factor governing the integration of environmental cues onto the epigenomic landscape.

## Results

### BAF60 represses hypocotyl elongation

**Fig. 1:**
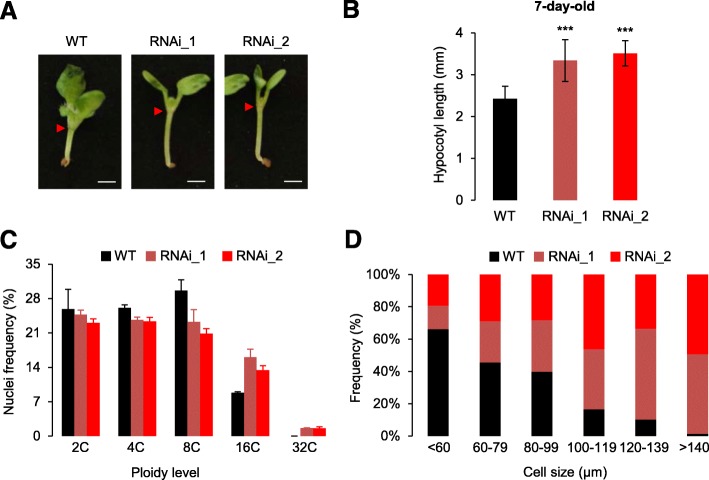
BAF60 represses hypocotyl elongation. **a** Seven-day-old wild-type (*WT*) and *Baf60* RNAi lines grown under LD conditions. The *red arrowheads* point to the top of the hypocotyl. *Bar* = 1 mm. **b** Hypocotyl length of 7-day-old wild type (WT) and *Baf60* RNAi lines (*RNAi_1* and *RNAi_2*) grown under LD conditions. Values are average ± standard deviation (n > 100). *Asterisks* indicate significantly different values (Student’s *t*-test, *P* < 0.05). **c** Ploidy level distribution of nuclei isolated from 14-day-old seedlings of the wild type (*WT*; *black*) and *Baf60* RNAi lines (*red*) grown in LD conditions. The y-axis shows the quantity of DNA in a haploid cell in G1. Data are average ± standard deviation from four independent experiments. **d** Cell length distribution of hypocotyls from 14-day-old wild type (*WT*; *black*) and *Baf60* RNAi lines (*red*) grown under LD conditions. For each sample, a total of 120 cells were measured

Because *BAF60* null alleles generate severe pleiotropic developmental defects [53], we used *Arabidopsis* RNA interference (RNAi) lines in which its expression is downregulated to analyze its influence on hypocotyl growth [47, 52, 54]. Under both long-day (LD) and short-day (SD) photoperiods, the hypocotyl length of the two tested independent RNAi lines was significantly longer than for wild-type seedlings (Fig. 1a, b; Additional file 1: Figure S1a, b). Reciprocally, two independent BAF60-CFP overexpressing lines displayed a significantly shorter hypocotyl compared to wild-type plants (Additional file 1: Figure S1c, d), suggesting that BAF60 could repress hypocotyl growth. This function was further tested upon growth at 28 °C or in the absence of light, conditions that trigger morphogenic responses characterized by enhanced hypocotyl elongation [1, 12]. Under such treatments, it was observed that *BAF60* RNAi lines displayed highly elongated hypocotyls at 28 °C (Additional file 1: Figure S1e), while in darkness the *BAF60* RNAi lines displayed wild-type etiolated phenotypes (Additional file 1: Figure S1f). In addition, two independent BAF60 overexpressing lines displayed significantly shorter hypocotyls than wild-type seedlings under dark condition (Additional file 1: Figure S1f). Altogether, these observations indicate that BAF60 negatively influences hypocotyl elongation under light and high temperature conditions.

Considering that hypocotyl growth is mostly driven by cell elongation [55, 56] and that hypocotyl cell size strongly correlates with endoreduplication levels, we quantified by flow cytometry the nuclear DNA content of hypocotyls dissected from wild-type and RNAi seedlings. In agreement with their long hypocotyl phenotype, the two *BAF60* RNAi lines displayed higher ploidy levels (Fig. 1c; Additional file 1: Figure S1g) and larger hypocotyl cells (Fig. 1d) compared to wild-type seedlings. Taken together, these observations show that BAF60 can repress hypocotyl elongation under light and high temperature conditions, possibly through the control of both cell elongation and endoreduplication.

### Regulation of *BAF60* gene expression by light and the circadian clock

**Fig. 2:**
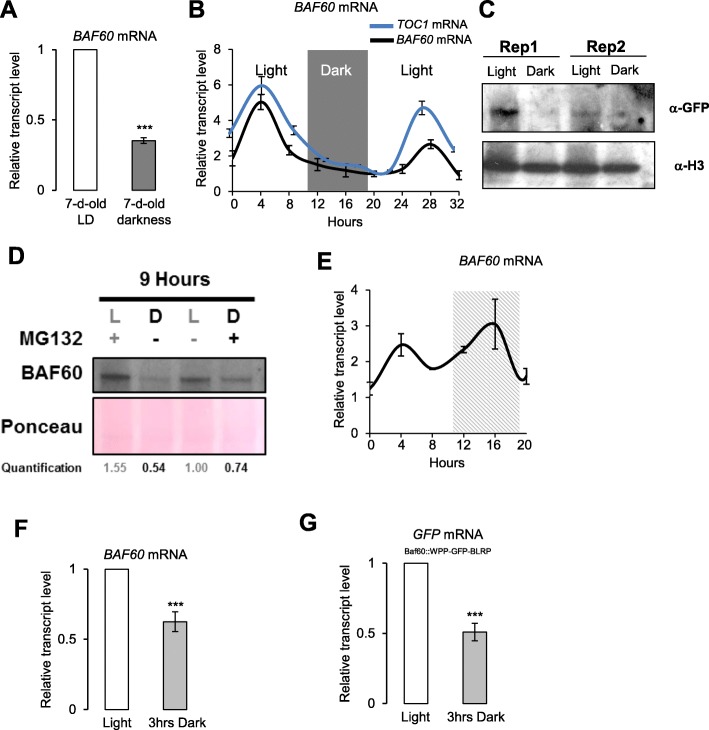
*BAF60* expression is regulated by both light and circadian rhythm. **a** RT-qPCR of *Baf60* expression in a 7-day-old wild-type line (Ws) grown in LD (*7-d-old LD*) or in darkness (*7-d-old darkness*) conditions. Values are average ± standard deviation obtained from three independent replicates. *Asterisks* indicate significantly different values (Student’s *t*-test, *P* < 0.05). **b** RT-qPCR data showing the relative expression of *Baf60* in wild-type plants under LD conditions in comparison to *TOC1*, a circadian clock-regulated gene. Total RNA samples were collected every 4 h from 14-day-old plants during a 32-h period (*Light* and *Dark* represent day and night periods, respectively). The *grey area* behind the trace represents the night period. Values are average ± standard deviation obtained from three independent replicates. **c** Immunoblot analysis showing the amount of BAF60 protein under light and dark conditions, obtained from a promBAF60::BAF60-CFP line. **d** Immunoblot analysis showing that MG132 can prevent BAF60 degradation through proteasome 26S in the same ratio under light and dark conditions. This result indicates that BAF60 degradation is independent of light. **e** RT-qPCR data showing the relative expression of *BAF60* in wild-type plants under continuous light. The seedlings were grown under LD conditions and were then transferred to continuous day conditions for the indicated period of time. Total RNA samples were collected every 4 h from 14-day-old plants during a 20-h period. The *grey area* behind the trace represents the period that would have corresponded to the night. Values are average ± standard deviation obtained from three independent replicates. **f** RT-qPCR quantification of *BAF60* expression in 7-day-old wild type seedlings grown in LD conditions and then transferred to darkness (*3hrs Dark*, *grey*) or kept in the light (*Light*, *white*) for 3 h. Values are average ± standard deviation and are representative of three biological replicates. *Asterisks* indicate significantly different values (Student’s *t*-test, *P* < 0.05). **g** *BAF60* promoter activity observed by RT-qPCR quantification of *GFP* expression in 7-day-old BAF60::WPP-GFP-BLRP seedlings grown in LD conditions and then transferred to darkness (*3hrs Dark*, *grey*) or not (*Light*, *white*) for 3 h. Values are average ± standard deviation obtained from three independent replicates. *Asterisks* indicate significantly different values (Student’s *t*-test, *P* < 0.05)

The observation that *BAF60* RNAi lines display a long-hypocotyl phenotype under photocycles but not in continuous darkness suggests that BAF60 could be differentially active under these conditions. To test this hypothesis, we quantified *BAF60* transcripts by RT-qPCR in light and dark-grown seedlings. Interestingly, *BAF60* RNA levels were lower in etiolated than in photomorphogenic seedlings (Fig. 2a; Additional file 1: Figure S2a). Regulation of the *BAF60* gene by light was further tested by monitoring its expression during a 32-h period: *BAF60* was more expressed during the day than during the night (Fig. 2b), in contrast to *ST2a*, which is mainly expressed during the night (Additional file 1: Figure S2b). Similarly, we assessed the BAF60 levels in a promBAF60::BAF60-CFP line through a western-blot assay, finding significantly higher levels of the protein during the daytime than in darkness (Fig. 2c). Furthermore, we assessed the stability/half-life of the BAF60 protein by measuring the level of the protein in dark and light conditions in the presence or absence of MG132, a proteasome inhibitor. The performed immunoblot analysis revealed that nuclear BAF60-CFP levels are increased by MG132 treatment in the same ratio in both dark and light conditions (Figs. 2d; Additional file 1: Figure S2c), suggesting that BAF60-CFP is degraded by the 26S proteasomes, but light-independently. However, we noticed that the decrease in *BAF60* gene expression occurred at dusk, before the beginning of the dark period (Fig. 2b). To investigate the potential role of the circadian clock on *BAF60* diurnal regulation, we performed a time-course analysis throughout a continuous light 20-h period after entrainment by long-day photocycles. Interestingly, *BAF60* was highly expressed during the period corresponding to the subjective night (Fig. 2e), indicating that light perception and the circadian clock may both contribute to fine-tune the expression of this gene. Finally, to confirm the potential effect of light on its expression, its transcript levels were measured upon a light-to-dark shift, finding, as expected, that *BAF60* mRNA level decreased after 3 h of darkness (Fig. 2f).

To test the potential direct effect of light on *BAF60* transcription, the *BAF60* promoter was cloned upstream of a green fluorescent protein (GFP) coding sequence and stably introduced into wild-type plants (BAF60::WPP-GFP-BLRP). *GFP* transcript levels were quantified upon transferring light-grown plants to darkness, showing a significant decrease after 3 h (Fig. 2g), similar to the phenomenon observed when measuring the expression levels of the *BAF60* locus through RT-qPCR. Comparison of *GFP* RNA levels between dark- and light-grown seedlings also showed more *BAF60* promoter activity in the light condition (Additional file 1: Figure S2d). Finally, analysis of public genome-wide data allowed us to determine that the *BAF60* promoter domain becomes more susceptible to DNase I footprinting during de-etiolation [29] (Additional file 1: Figure S2e), corroborating its enhanced transcriptional activity in response to light. Taken together, these results indicate that *BAF60* expression is regulated at the transcriptional level, and possibly also at the post-transcriptional level, by light and the circadian clock.

### BAF60 binds nucleosome-free regions of expressed genes

**Fig. 3:**
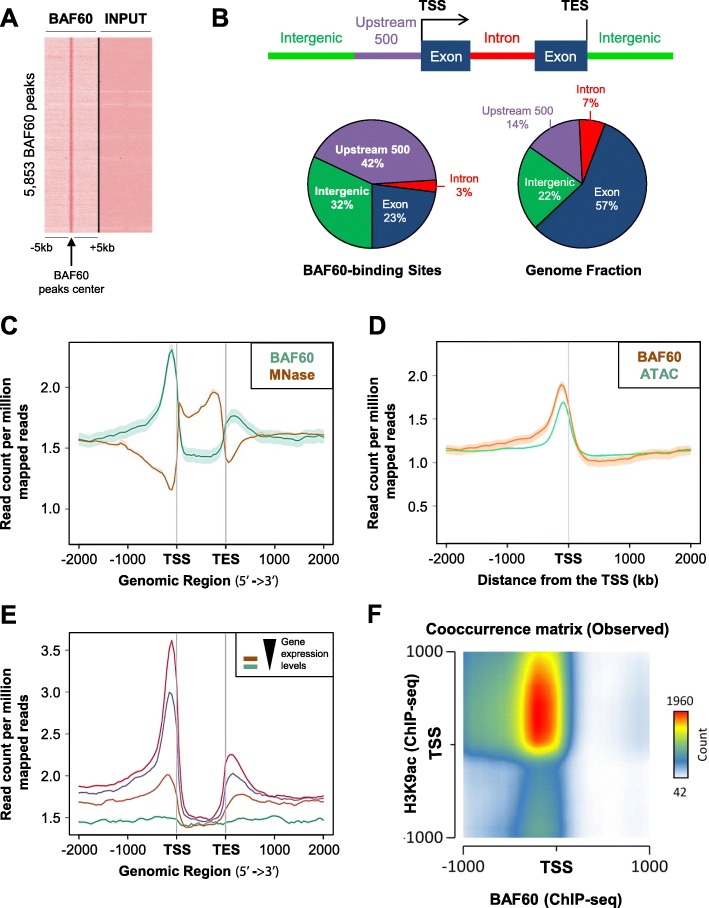
BAF60 binds nucleosome-free regions of transcribed genes. **a** Comparison between BAF60 and input of tag density in the region ±5 kb around the BAF60 peaks. ChIP-seq was performed on 14-day-old BAF60-CFP overexpressing plants (OE BAF60-CFP_1) grown under LD conditions. **b** Pie chart representation of the distribution of BAF60 peaks identified by ChIP-seq in four different genomic regions. The definition of each region is described above. *TES* transcription end site, *TSS* transcription start site. **c** Mean profile of BAF60 ChIP-seq and MNase-seq read density with respect to a gene model from TSS to TES. Normalization of coverage using spline algorithm was performed over the genes and flanking 2-kb region. **d** Merged profiles of BAF60 ChIP-seq and ATAC-seq read density over TSS and flanking 2-kb region. **e** Average enrichment profile of BAF60 is correlated with gene expression variation. Gene expression is categorized from low (first quantile) to high (fourth quantile) expression. Mean-normalized ChIP-seq densities of equal bins along the gene and 2-kb region flanking the TSS or the TES are plotted. Highly expressed genes show higher enrichment for binding of BAF60. **f** Co-ocurrence matrix representing the colocalization of H3K9ac marks and BAF60 binding along the BAF60 targets

In order to assess how BAF60 influences seedling development, we explored the genomic loci at which BAF60 could exert its activity by chromatin immunoprecipitation-sequencing (ChIP-seq) of BAF60-CFP, using an anti-GFP antibody in light-grown plants. Through this method we identified a wide repertoire of 5853 BAF60-CFP binding peaks (Fig. 3a) corresponding to 3729 genes. The large majority (74%) of BAF60 binding sites corresponded to intergenic regions and to 500-bp domains upstream of transcription start sites (TSSs), which usually correspond to promoter regions (Fig. 3b). Interestingly, the average profile of all mapped reads over a gene model further revealed that BAF60 is enriched over both the 5′ and 3′ ends of genes, confirming our previous studies [47, 50–52] (Additional file 1: Figure S3). Because it has been previously reported that BAF60 is a component of plant CRCs [46], the relationship between nucleosome regions and BAF60-enriched loci was explored by mapping MNase hypersensitive sites using MNase-seq assay on wild-type plants. Through this method we found that the BAF60-CFP peaks largely anti-correlate with nucleosomal occupancy in wild-type seedlings (Fig. 3c). Furthermore, we performed an ATAC-seq (assay for transposase-accessible chromatin using sequencing), a technique that allows precise positioning of nuclesosome-free regions (NFRs) [57]. Interestingly, this revealed a perfect correlation between NFR profiles and BAF60 positioning around the TSSs (Fig. 3d). Altogether, these analyses uncovered that BAF60 is frequently enriched over the 5′ NFR of hundreds of genes, in agreement with a potential direct influence of BAF60 on local transcriptional controls.

To understand the relationship between BAF60 and gene expression, we tested how its enrichment relates to mRNA levels. This revealed a positive correlation between the binding frequency of this protein and the number of transcripts of specific loci (Fig. 3e). We also analyzed the relationship between gene expression and DNA accessibility in a genome-wide fashion, finding that the most expressed genes display a highest level of accessibility in their 5′ and 3′ ends compared to less transcribed genes (Additional file 1: Figure S4). Finally, to determine whether BAF60 associates with specific chromatin contexts, we compared its genome-wide positioning with publically available epigenomic profiles of histone modifications. This showed that more than 50% of the BAF60 target genes are marked by histone 3 lysine 9 acetylation (H3K9ac), confirming that BAF60 frequently binds active chromatin regions (Fig. 3f). On the other hand, BAF60 binding co-localizes, but to a much lower extent, with the repressive marks H3K27me3 and H3K9me2, not only in the gene body but also in the promoter regions, upstream of the TSS (Additional file 1: Figure S5).

### BAF60 targets G-box motifs and acts antagonistically to PIF4

**Fig. 4:**
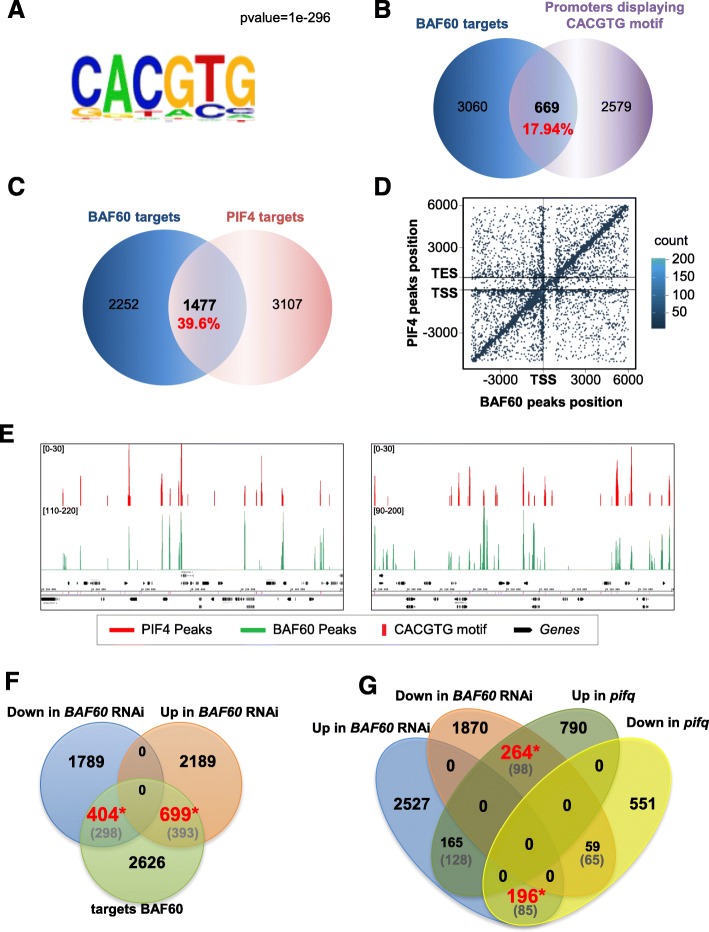
BAF60 preferentially associates with G-box motifs and shares a large repertoire of target genes with PIF4. **a** HOMER motif search identifies a major BAF60-associated motif defined as the G-box (CACGTG). *P* value = 1e-296. **b** Venn diagram representing the overlap between BAF60 target genes and genes containing a G-box motif in their promoter (500-bp upstream domains). **c** Venn diagram representing the overlap between BAF60 and PIF4 target genes. **d** Density plot showing overlap of PIF4 and BAF60 using hexagonal binning routine. As a large number of data points may overlap, hexagonal binning gives an additional dimension of differentiation of overlapping points based on count. Each point represents the distance of the midpoint of a peak to the nearest gene. On the *y-axis* is the location of the midpoint of a PIF4 peak in comparison to gene position; on the *x-axis* is the location of a midpoint of BAF60 in comparison to the nearest gene. A large number of points occurs along the positive correlation line, showing the co-occurrence pattern of PIF4 and BAF60. **e** Genome Browser snapshots of BAF60 (*green*) and PIF4 (*red*) ChIP-seq peaks on two representative genomic regions of chromosome 4 [chr4:10,235,000–10,361,000] (*left*) and chromosome 1 [chr1:28,285,000–28,381,000] (*right*). Genes are shown in *black* and G-boxes in *pink*. **f** Venn diagram representing the overlap between the BAF60 targets, found through ChIP-seq, and the missregulated genes in the *Baf60* RNAi lines. The values in parentheses (*grey*) correspond to the common elements expected by chance, while those in *black* or *red* represent the observed results. The values in *red* represent significant enrichment, while those in *black* are not significantly different from those expected by chance (Chi-squared test). **g** Venn diagram representing common missregulated genes in the *Baf60* RNAi line and the *pifq* mutant. The values in parentheses (*grey*) correspond to the common elements expected by chance, while those in *black* or *red* represent the observed results. The values in *red* represent significant enrichment, while those in *black* are not significantly different from those expected by chance (Chi- squared test)

Given that BAF60-CFP distribution is enriched over NFRs, we hypothesized that BAF60-associated CRCs could recognize specific DNA-sequence motifs rather than chromatin signatures. De novo motif discovery using the HOMER software identified the G-box consensus sequence (CACGTG) as an over-represented *cis* element within the BAF60 peaks (Fig. 4a; Additional file 1: Figure S6a). This motif is known to recruit multiple transcription factors such as PIF4 [58]. Using the known repertoire of PIF4-binding sites identified by ChIP-seq using *pifQ*/*pPIF4*::*PIF4myc* transgenic plants [58], we firstly found that 17.96% of PIF4 targets displayed a G-box motif positioned 500 bp upstream of the TSS (Additional file 1: Figure S6b). Interestingly, the same analysis performed with BAF60-CFP peaks identified that 17.94% of the BAF60 targets (669 targets out of 3730) also contain a CACGTG motif over the 500-bp upstream domains (Fig. 4b). Furthermore, direct comparison of the PIF4 and BAF60 target genes identified a large overlap, 39.6% of BAF60 targets with PIF4 targets, suggesting that these two proteins regulate common genes (Fig. 4c). Accordingly, dot-plot analysis of the precise BAF60-CFP and PIF4 peaks along gene structures further showed frequent clustering along the same positions (Fig. 4d). Finally, visual inspection of BAF60-CFP and PIF4 peaks confirmed their similar distributions over many genomic positions, the majority of which correspond to G-box motifs (Fig. 4e). A gene ontology analysis, using the agriGO tool, revealed that there is a significant enrichment of genes involved in the response to light stimuli, to far red and blue light, and to temperature in the common targets of BAF60 and PIF4 (Additional file 1: Figure S7). Altogether, these analyses uncover a potential role of BAF60 at hundreds of PIF4 binding sites that represent targets for both proteins, involved in the regulation of photomorphogenesis and heat responses.

To understand the impact of BAF60 on gene expression we performed a transcriptomic analysis of BAF60 RNAi lines and found that 1103 of the BAF60 targets displayed missregulation in both of the *BAF60* RNAi lines, 404 downregulated and 699 upregulated (Fig. 4f). This result suggests that BAF60 regulates either positively or negatively the expression of several of its targets. A gene ontology analysis of the BAF60 target genes that are upregulated in both RNAi lines revealed that there is a significant enrichment of genes involved in the response to light stimuli and hormone-mediated signaling (Additional file 1: Figure S8).

**Fig. 5:**
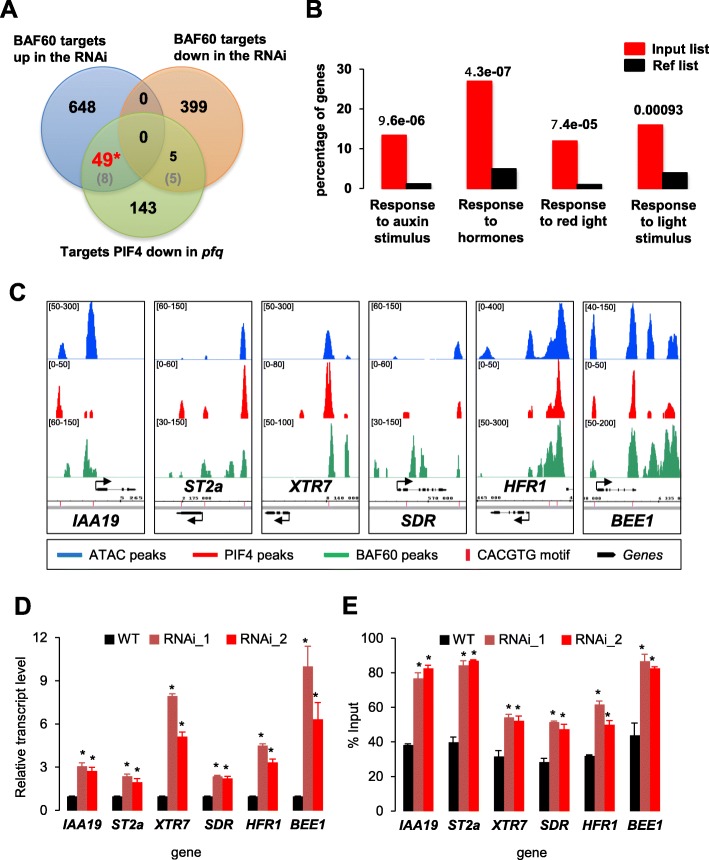
BAF60 has opposite effects than PIF4 on the expression of hypocotyl elongation regulatory genes. **a** Venn diagram displaying the elements in common between the BAF60 targets missregulated in the *Baf60* RNAi line and the PIF4 targets downregulated in the *pifq* mutant. The values in parentheses (*grey*) correspond to the common elements expected by chance, while those in *black* or *red* represent the observed results. The values in *red* represent significant enrichment, while those in black are not significantly different from those expected by chance (Chi2 square test). **b** Gene ontology analysis of the common targets oppositely regulated by PIF4 and BAF60. The *red bars* represent the input and the *black bars* the reference. This group of loci is significantly enriched in genes involved in response to auxin, hormones, and light. **c** Genome Browser snapshots of ATAC-seq, BAF60, and PIF4 ChIP-seq peaks on six common target genes (*IAA19*, *ST2a*, *XTR7*, *SDR*, *HFR1*, and *BEE1*). BAF60-associated peaks are shown in *green*, PIF4-binding peaks in *red*, ATAC-seq peaks in *blue*, annotated genes in *black*, and G-box motifs in *pink*. **d** RT-qPCR analysis showing the relative expression of the indicated genes in 7-day-old seedlings in LD conditions. Values are average ± standard deviation obtained from three independent replicates and *asterisks* represent significant difference from the wild type (*WT*; Student’s *t*-test, *P* < 0.05). **e** DNA accessibility measured by FAIRE-qPCR in 7-day-old wild-type and *BAF60* RNAi seedlings grown in LD conditions. Higher values correspond to more accessible DNA. Primer pair 1 was used for *ST2a*, *SDR*, and *HFR1* loci, pair 3 for *IAA19* and *BEE1* loci, and pair 2 for the *XTR7* locus. *Error bars* represent the standard deviation from three biological replicates and asterisks represent significant difference from the WT (Student’s *t*-test, *P* < 0.05)

Having shown that BAF60 and PIF4 bind to hundreds of common genes, we compared the RNA-seq data obtained from *BAF60* RNAi lines and a *pifq* mutant, finding that a representative group of genes displayed opposite transcriptomic profiles; 264 genes are downregulated in *BAF60*-*RNAi* lines but upregulated in *pifq*, while 196 are upregulated in *BAF60*-*RNAi* lines but downregulated in *pifq* (Fig. 4g). In addition, from the shared BAF60 and PIF4 targets, 49 genes are upregulated in the *BAF60* RNAi line and downregulated in the *pifq* mutant (Fig. 5a). In all these cases the number of enriched genes is greater than what would be expected by chance, supporting the antagonistic function of the two proteins regarding the expression of this set of genes. Interestingly, a gene ontology analysis revealed that, of the 49 oppositely regulated targets in the two accessions, a significant group is involved in processes such as response to hormones (notably auxin), to light, and more specifically, to red light (Fig. 5b).

Given the prominent role of PIF4 and the negative effect of BAF60 on hypocotyl elongation, we analyzed in detail the functional relationship of BAF60 with cell size regulatory genes that impact this process. These were found to be targeted by both PIF4 and BAF60 and upregulated in the *BAF60* RNAi line and downregulated in the *pifq* mutant (*IAA19*, *ST2a*, *XTR7*, *SDR*, *HFR1*, and *BEE1*). Our ChIP-seq and ATAC-seq analyses indicated that both BAF60 and PIF4 target the promoter and/or the gene body of these genes around a G-box motif at loci with high DNA accessibility (Fig. 5c). Targeted ChIP-qPCR further confirmed that BAF60-CFP is enriched over these loci (Additional file 1: Figure S9). To confirm its influence on their expression, we quantified the transcripts of these genes upon knocking down *BAF60*. RT-qPCR analysis showed highly increased mRNA levels in the two *BAF60* RNAi lines compared to wild-type seedlings for the six genes (Fig. 5d). Consistently, expression of these genes was reduced in overexpressing (OE) lines (Additional file 1: Figure S10a). Altogether, these observations indicate that BAF60 represses these genes in *cis*.

**Fig. 6:**
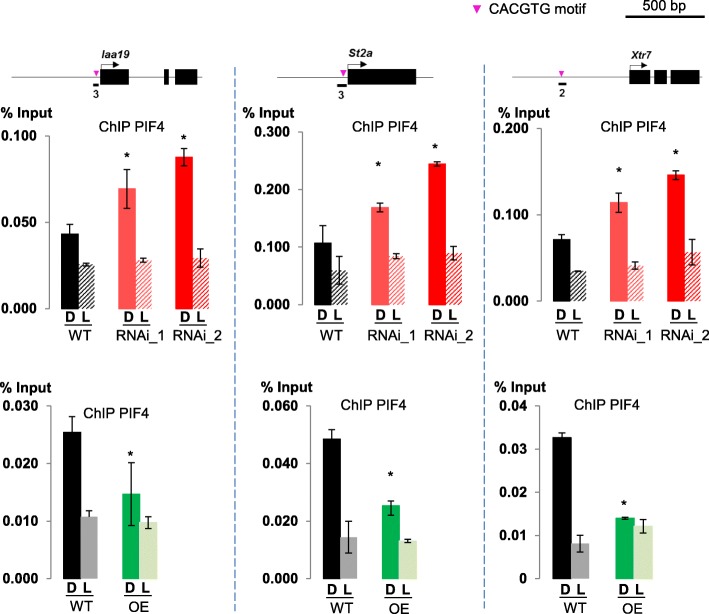
BAF60 and PIF4 actively compete in vivo for their targets. Seven-day-old plantlets of the two BAF60 RNAi lines, a BAF60 OE line, and the corresponding WT (WS and Col0, respectively) were used to analyze PIF4 binding on selected target genes (*IAA19*, *ST2A*, and *XTR7*) by ChIP-qPCR. PIF4 binding to targets is increased in the darkness in the *BAF60* RNAi lines in comparison with the WT. On the contrary, its binding was diminished in the BAF60 OE line, indicating that BAF60 and PIF4 compete for their targets and that changes in BAF60 levels affect PIF4 binding. Data are average of three technical replicates ± standard deviation. *Asterisks* indicate significantly different values (Student’s *t*-test, *P* < 0.05). *D* dark, *L* light

Given the implication of BAF60 in modulating histone composition and occupancy at the *FLC* locus [47], as well as its enrichment over the *IAA19*, *ST2a*, *XTR7*, *SDR*, *HFR1*, and *BEE1* genes, we proposed that the BAF60 CRC may potentially repress transcription of these genes through nucleosome remodeling. We therefore tested the effect of knocking down *BAF60* on local chromatin accessibility, using a targeted formaldehyde-assisted isolation of regulatory elements assay (FAIRE) [59]. Interestingly, this approach showed that the promoter region of the six tested genes displayed a more open conformation in the two analyzed *BAF60* RNAi lines compared to wild-type seedlings (Fig. 5e). On the other hand, and coherently, the OE line presented reduced promoter accessibility in the studied genes in darkness, reaching levels similar to those of the WT under light conditions (Additional file 1: Figure S10b). Taken together, these analyses indicate that BAF60 antagonizes the role of PIF4 in the expression of genes controlling hypocotyl elongation by decreasing their accessibility and thereby repressing their transcription. Furthermore, through a ChIP-qPCR assay, we found that, in the *BAF60* RNAi lines, the enrichment of PIF4 at its target loci is increased, in contrast to the BAF60 OE lines, where PIF4 binding is reduced (Fig. 6). These results indicate that BAF60 and PIF4 actively compete in vivo for their targets.

## Discussion

Several chromatin modifications such as histone methylation and acetylation have recently been shown to influence genome expression reprogramming during photo- and thermomorphogenesis [12, 21, 22]. Still, the role of chromatin remodeling in the environmental control of seedling morphogenesis is poorly understood. In this study, we found that the SWI/SNF CRC subunit BAF60 is required for efficient repression of hypocotyl elongation under light and high temperature conditions by repressing cell elongation and endoreduplication, acting on local chromatin status of cell size regulatory genes. Hypocotyl elongation relies only on cell growth and not on cell division; in hypocotyl cells, DNA replication leads to 4C and 8C ploidy levels in light- and dark-grown seedlings, respectively [55], a control of DNA content that is thought to be associated with cell size regulation [55, 56]. Here, we first observed that inhibition of endoreduplication by light in the hypocotyl requires BAF60. This adds to our former identification of BAF60 as a protein partner of KRP5, a cell cycle inhibitor that promotes endoreduplication and hypocotyl elongation [60]. The functional significance of this interaction is still elusive, but KRP5 could hypothetically interact with BAF60 to reduce its activity under environmental conditions that enhance hypocotyl elongation, such as darkness and high temperature.

This work also unveiled that the expression of *BAF60* itself is regulated at the transcriptional level. Greater accessibility of the *BAF60* promoter during de-etiolation detected in DNase I footprinting datasets [29] is a good in vivo indication of enhanced transcriptional activity in response to light signals. Consistently, we confirmed that its promoter domain drives more transcription under light than under dark conditions using a *pBAF60*::*WPP*-*GFP*-*BLRP* transcriptional reporter.

Interestingly, this combination of regulatory events tightly control BAF60 protein level in response to both light and circadian signals. Circadian rhythms are set by diurnal fluctuations of light and temperature signals [61, 62]. In addition, clock-controlled transcriptional regulation is highly integrated with environmental signals such as light and temperature, notably converging onto a common repertoire of genes that usually exert their function in specific light conditions or daytime [63]. Clock- or light-controlled changes in BAF60 abundance might be directly linked to its function. Accordingly, *BAF60* downregulation strongly enhances hypocotyl elongation under photocycles. Given that BAF60 triggers the repression of *IAA19*, *ST2a*, *XTR7*, *SDR*, *HFR1*, and *BEE1* genes, low *BAF60* expression levels at night, or upon a shift to darkness, could therefore mediate direct control of these cell size regulatory genes when plants face these conditions.

Genome-wide profiling of nucleosome positioning has recently been performed in a number of eukaryotic organisms, showing that gene bodies have high nucleosome occupancy [64, 65]. In contrast, regulatory regions located in promoters or terminators tend to have low nucleosome occupancy and often contain a NFR. Recently, the INO80 and SWR1 chromatin remodeling complexes have been found to target NFR regions to regulate histone variant deposition [66]. Using a combination of genome-wide methodologies (ChIP-seq, ATAC-seq, and MNase-seq), we found that BAF60 binds hundreds of NFRs, showing that, like in the yeast *Saccharomyces cerevisiae*, plant chromatin remodelers can target nucleosome-free regions. The position of the nucleosomes along a particular DNA domain and the presence of a NFR can have profound effects on its accessibility and on its transcriptional regulation by *trans*-acting factors [67]. Nevertheless, these results do not imply that BAF60 acts on its targets as a transcriptional activator, but rather suggest that BAF60 regulates the expression of genes with a high expression level. As it was recently shown for the catalytic subunit BRM, BAF60 can function as both an activator or inhibitor of gene expression [67]: genes that were upregulated in BAF60 RNAi lines and genes that were downregulated were both significantly enriched amongst BAF60 targets. In the case of cell size regulatory genes, our analyses by FAIRE-qPCR showed that DNA accessibility around NFRs was increased in *BAF60* RNAi lines, which at the same time is correlated with increased gene expression. These findings are highly consistent with the local association of BAF60 protein with these domains in vivo and with its role as a negative regulator of their expression.

Other proteins might facilitate the recruitment of BAF60 to such NFRs. Indeed, the SWI/SNF chromatin remodeler of *Arabidopsis*, BRAHMA (BRM), was reported to be recruited by the H3K27 demethylase REF6 to specific sequences. The demethylase was found to bind its target motifs through their recognition by zinc-finger (ZnF) domains, facilitating BRM targeting [68]. A similar mechanism may be occurring in the case of BAF60, and for future studies we will consider determining its DNA-binding protein interactors, which would possibly provide evidence on the existence of other molecules involved in the recruitment of BAF60 to its targets within NFRs.

Determining the distribution of BAF60 over the genome led us to identify its preferential enrichment over G-box sequence motifs. Interestingly, this motif is also recognized by PIF transcription factors [11]. Consistently, we observed that BAF60 and PIF4 share 1477 target genes. Several PIF family members (PIF1, PIF3, PIF4, and PIF5) play central roles in the repression of hypocotyl elongation in darkness [69]. Interestingly, PIF4 is also central for integrating light and high temperature signaling and for mediating their influence on seedling growth [12]. In contrast to PIF4, *BAF60* knockdown lines display a long hypocotyl phenotype under light and high temperature. This suggests that BAF60 and PIF4 antagonistically regulate a common set of genes. Furthermore, in our study, BAF60 and PIF4 were found to compete for their targets, a similar mechanism of PIF4 regulation to the one reported by Gangappa and Kumar [20]. PIF4 activity appears to be regulated and antagonized by various factors, including BAF60 and HY5, which compete with PIF4 for binding to several targets, including cell size regulatory genes. Consistently, increased expression of *IAA19*, *ST2a*, *XTR7*, *SDR*, *HFR1*, and *BEE1* genes in the *BAF60* RNAi lines correlated with higher accessibility of their promoter domains. The observation that the 5′ regions of genes became hypersensitive to micrococcal nuclease upon gene activation in *Drosophila* was among the earliest demonstrations of this phenomenon [70–73]. The appearance of hypersensitive sites reflects a loss or a destabilization of nucleosomes at the promoters of transcribed genes [67]. Several mechanisms are thought to act in concert to achieve this, and can involve SWI/SNF CRCs [74–76]. Hence, BAF60 could bind NFRs of genes involved in hypocotyl growth control to negatively regulate their transcription through the modulation of DNA accessibility.

## Conclusions

**Fig. 7:**
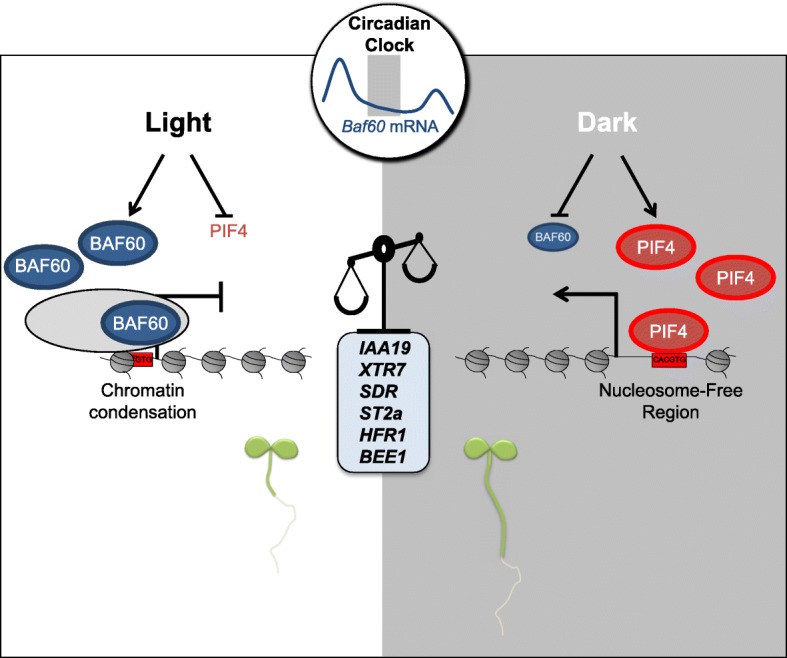
Working model for hypocotyl elongation control by BAF60 chromatin remodeling. BAF60 is more abundant under light conditions and represses expression of hypocotyl elongation regulatory genes, *IAA19*, *ST2a*, *XTR7*, *SDR*, *HFR1*, and *BEE1*, upon recruitment onto G-box motifs by modulating DNA accessibility. In darkness and at nighttime the abundance of BAF60 decreases, possibly allowing PIF proteins to bind the same regulatory loci and promote expression of the target genes. However, remaining BAF60 protein competes with PIF4 for binding on their common targets, thereby fine-tuning the expression level of genes involved in cell size control

These findings allow us to propose a model in which BAF60 acts under the control of light signaling pathways to repress G-box-containing genes involved in seedling morphogenesis. In the absence of light perception, BAF60 protein levels decrease dramatically, allowing PIF proteins to bind and promote the expression of BAF60 targets (Fig. 7). Transcriptional reprogramming in response to light signals involves massive changes of chromatin states along the genome [27–30], and BAF60 appears to be an important player in this reorganization. More generally, this study raises the possibility that SWI/SNF complexes are key factors governing the environmental control of plant adaptive responses.

## Methods

### Plant material and growth conditions

*Arabidopsis thaliana* seeds of both RNAi lines *BAF60*_*1* (CS30982) and *BAF60*_*2* (CS23961), in the Wassilewskija (Ws) background, were obtained from the Nottingham *Arabidopsis* Stock Centre (NASC). Plant lines overexpressing BAF60 35S::BAF60-CFP_1 and 35::BAF60-CFP_2 have been described previously [47]. BAF60::WPP-GFP-BLRP, 35S::GFP plants were in the Columbia-0 background (Col0). Plants were grown in chambers at 20 °C on sterile half-strength MS medium and 0.8% agar under long days (16 h of light at 20 °C, 8 h of darkness at 18 °C; LD), short days (8 h of light at 20 °C, 16 h of darkness at 18 °C; SD) or darkness conditions (16 h of darkness at 20 °C, 8 h of darkness at 18 °C; D). Seeds were surface-sterilized by treatment with bayrochlore for 20 min, washed, and imbibed in sterile-water for 2–4 days at 4 °C to obtain homogeneous germination. Phenotyping under high temperature was performed by cultivating plants under continuous light at 23 °C during 3 days and then at 28 °C for 4 days. For hypocotyl growth assays, plates were scanned and hypocotyl length was measured using the Image J software (http://rsb.info.nih.gov/ij/).

### Flow cytometry

Fourteen-day-old hypocotyls were chopped with a razor 587 blade in 1 mL of Galbraith buffer supplemented with 1% polyvinylpyrrolidone 10,000, 5 mM metabisulfite, and 5 mg/mL RNase from a stock solution at 50 units/mg. Propidium iodide was added to the filtered supernatants at a final concentration of 50 μg/ml. Endoreduplication levels of 5000–10,000 stained nuclei were determined using a Cyflow SL flow cytometer (Partec) with a 532 nm solid state laser (30 mW) excitation and emission collected after a 630/30 nm filter.

### Cell size measurement

For cell size measurement, hypocotyls of 14-day-old plantlets were fixed in ethanol:acetic acid (3:1) and washed in 70% (v/v) ethanol during 20 min at room temperature. Plantlets were subsequently cleared with chloral hydrate (8 g of chloral hydrate [Sigma], 2 mL of 50% glycerol [w/v], and 1 mL of water) overnight. The day after, samples were mounted on slides in water under coverslips, and differential interference contrast macroscopy (AZ100; Nikon) was used to capture images with a Nikon RI1 video camera. Cell size measurement was performed with the ImageJ software.

### RNA extraction and real-time quantitative PCR analysis

Total RNAs were extracted from 180 mg of seedlings with the RNeasy MiniPrep kit (Qiagen) for RT-qPCR or with the ZR Plant RNA MiniPrep kit (Zymo Research) for RNA-seq, according to the manufacturer’s instructions. First strand cDNA was synthesized from 2 μg of total RNAs using Improm-II reverse transcriptase (A3802, Promega) according to the manufacturer’s instructions. We mixed 1/25th of the synthesized cDNA with 500 nM of each primer and LightCycler® 480 Sybr Green I master mix (Roche Applied Science) for quantitative PCR analysis (qPCR). Products were amplified and fluorescent signals acquired with a LightCycler® 480 detection system. The specificity of amplification products was determined by melting curves. *Ubq10* was used as an internal control for signal normalization. The Exor4 relative quantification software (Roche Applied Science) automatically calculates relative expression levels of the selected genes with algorithms based on the ΔΔCt method. Data were from duplicates of at least three biological replicates. All primer sequences used are given in Additional file 1: Table S1.

### Formaldehyde-assisted isolation of regulatory elements assay

Formaldehyde-assisted isolation of regulatory elements (FAIRE) was performed as described by Ariel et al. [59]. Two grams of 7-day-old seedlings were crosslinked in 1% (v/v) formaldehyde at room temperature for 15 min. Purified nuclei were resuspended in 500 μl, but only 50 μl were used (diluted to 500 μl) to continue with the protocol.

### Chromatin immunoprecipitation assay

ChIP-seq assays were performed on 14-day-old BAF60-CFP overexpressing line 1 (OE BAF60-CFP_1), formerly described by Jegu and collaborators [47], using an anti-GFP antibody (Clontech 632592), and on 14-day-old wild type plant (Col0) using an anti-H3K9ac antibody (Millipore), an anti-H3K9me2 antibody (Abcam), and an anti-H3K27me3 antibody (Millipore). ChIP-qPCR assays were performed on 7-day-old BAF60-CFP overexpressing line 2 (OE BAF60-CFP_2) using an anti-GFP antibody (Clontech 632592) and an anti-IgG antibody (Millipore). The ChIP protocol was modified from Gendrel et al. [77]. Briefly, after plant material fixation in 1% (v/v) formaldehyde, tissues were homogenized and nuclei isolated and lysed. Cross-linked chromatin was sonicated using a water bath Bioruptor UCD-200 (Diagenode, Liège, Belgium; 15 s on/15 s off pulses; 15 times). The complexes were immunoprecipitated with antibodies overnight at 4 °C with gentle shaking and incubated for 1 h at 4 °C with 50 μL of Protein AG UltraLink Resin (Thermo scientific). The beads were washed for 6 × 5 min in ChIP Dilution Buffer (SDS 0.01%, Triton X-100 1.1%, 1.2 mM EDTA, pH 8, 16.7 mM Tris-HCl, pH 8, and 167 mM NaCl) and twice in TE. ChIPed material was eluted by two 15-min incubations at room temperature with 250 μL elution buffer (SDS 1%, 0.1 M NaHCO3). Chromatin was reverse-crosslinked by adding 20 μL of NaCl 5 M and incubated overnight at 65 °C.

For qPCR, reverse-crosslinked DNA was then recovered using the IPure kit (Diagenode, Liège, Belgium) and analyzed by RT-qPCR. An aliquot of untreated sonicated chromatin was processed in parallel and used as the total input DNA control.

For ChIP-seq, reverse-crosslinked DNA was submitted to RNase and proteinase K digestion and extracted with phenol-chloroform. DNA was ethanol precipitated in the presence of 20 μg of glycogen and resuspended in 50 μL of H20 in a siliconized tube. IP or input DNA (10 ng) was used for ChIP-seq library construction using a NEBNext® Ultra DNA Library Prep Kit for Illumina®.

### Microccocal nuclease sensitivity assay

Five grams of 14-day-old seedlings were ground and nuclei were isolated with 4 °C buffer (0.25 M sucrose, 10 mM Tris-HCl, 10 mM MgCl_2_, 1% Triton, 5 mM β-mercaptoethanol) containing proteinase inhibitor cocktail (Roche), filtered with a 63 μm filter, and incubated in MN buffer (20 mM Tris-HCl, 70 mM NaCl, 20 mM KCl, 5 mM MgCl_2_, 3 mM CaCl_2_). MNase (100 U; Invitrogen®) was added to initiate the kinetics. Adding EGTA and EDTA to a final concentration of 2 mM stopped the reaction. DNA was extracted with phenol/chloroform, precipitated with isopropanol, and resuspended in ultra-pure water. Purified DNA was run on a 1% agarose gel and the band corresponding to mononucleosome was excised and purified with a MinElute Gel Extraction Kit (Qiagen). Mononucleosome DNA (10 ng) was used for ChIP-seq library construction using a NEBNext® Ultra DNA Library Prep Kit for Illumina®.

### Assay for transposase-accessible chromatin with high-throughput sequencing

Fourteen-day-old seedlings (100 mg) were ground and nuclei were isolated with 4 °C buffer (0.25 M sucrose, 10 mM Tris-HCl, 10 mM MgCl_2_, 1% Triton, 5 mM β-mercaptoethanol) containing proteinase inhibitor cocktail (Roche) and filtered with a 63 μm filter. Nuclei were resuspended in 1× TD buffer (Illumina FC-121-1030) and 2.5 μL of Tn5 transposes (Illumina FC-121-1030) were added. The transposition reaction was performed at 37 °C for 30 min, and DNA was purified using a Qiagen MinElute Kit. DNA libraries were amplified for a total of eight cycles as described by Buenrostro et al. [57].

### Deep sequencing and bioinformatics

Single-end sequencing of ChIP samples was performed using Illumina GAIIx with a read length of 50 bp. Reads were quality controlled using FASTQC (http://www.bioinformatics.babraham.ac.uk/projects/fastqc/). Trimmomatic was used for quality trimming. The reads were mapped onto the TAIR10 assembly using Bowtie [78] with mismatch permission of 1 bp. Unique mapping of reads was adopted. To identify regions that were significantly enriched, we used MACS2 [79]. Visualization and analysis of genome-wide enrichment profiles were done with IGB. Peak annotations, such as proximity to genes and overlap on genomic features such as transposons and genes, were assigned using HOMER. NGSplot was used to profile the enrichment of this mark at transcriptional start sites (TSSs) and along the gene [80]. To identify regions that were significantly enriched in histone modification data (H3K27me3), we used SICER [81] with parameters of W:200 (window length) and G:200 (gap size) for H3K9ac, and W:200, G:600 for H3K27me3 and H3K9me2. Nucleosome occupancy and shift between the wild type and BAF60 mutant were deduced from the MNase sequencing data using DANPOS [82]. DiffReps was used to find the differential marking between two histone modifications [83]. Spatial binding of the two peaks of BAF60 and PIF4 were done by position-wise comparison using a binning approach and plotted in hexplot.

### Accession numbers

BAF60 (AT5G14170), IAA19 (AT3G15540), ST2A (AT5G07010), XTR7 (AT4G14130), SDR (AT5G02540), HFR1 (AT1G02340), BEE1 (AT1G18400).

## Additional file

Additional file 1: Figures S1–S12 and Table S1. (PPTX 5057 kb)
