# Supplementary material for: The Arabidopsis SWI/SNF protein BAF60 mediates seedling growth control by modulating DNA accessibility
Source: Genome Biol. 2017 Jun 15;18:114. doi: 10.1186/s13059-017-1246-7 (PMC5471679; doi:10.1186/s13059-017-1246-7)
Supplement: Additional file 1: Figures S1–S12 and Table S1. — (PPTX 5057 kb) [file 13059_2017_1246_MOESM1_ESM.pptx]

## Slide 1
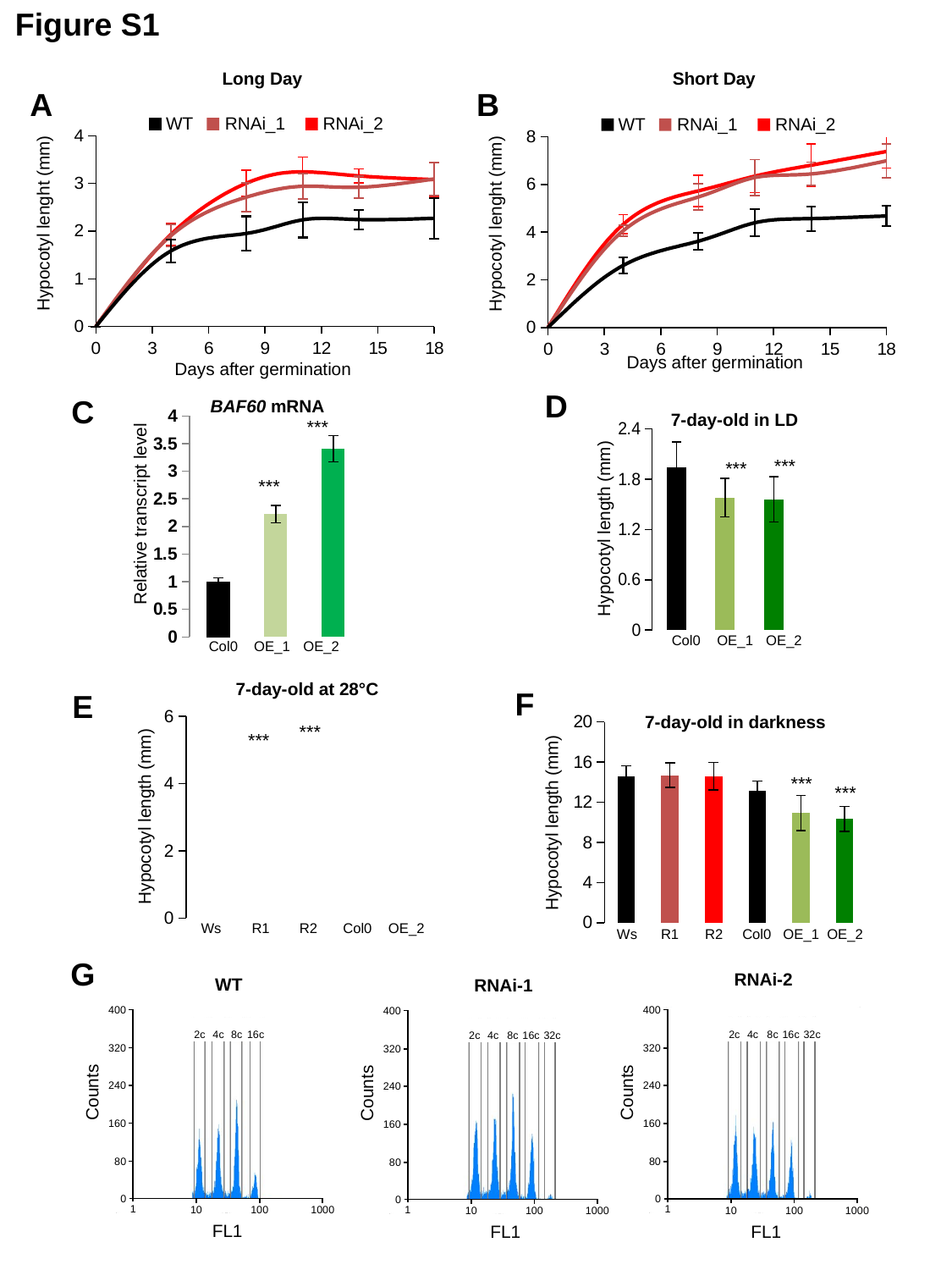

Figure S1
Short Day
WT
RNAi_1
RNAi_2
### Chart
| Category | | | |
|---|---|---|---|Hypocotyl lenght (mm)
Days after germination
Long Day
A
B
WT
RNAi_1
RNAi_2
### Chart
| Category | | | |
|---|---|---|---|Hypocotyl lenght (mm)
Days after germination
D
C
BAF60 mRNA
### Chart
| Category | |
|---|---|7-day-old in LD
### Chart
| Category | |
|---|---|***
***
Hypocotyl length (mm)
Col0
OE_1
OE_2
***
***
Relative transcript level
Col0
OE_1
OE_2
7-day-old at 28°C
### Chart
| Category | 28°C smallest plants removed (mm) |
|---|---|
| Ws | 2.786515834366776 |
| RNAi-1 | 4.275619309760745 |
| RNAi-2 | 4.687169172136354 |
| Col-0 | 1.785588256051945 |
| OE_2 | 1.974380690239255 |***
***
Hypocotyl length (mm)
Ws
R1
R2
Col0
OE_2
F
E
7-day-old in darkness
### Chart
| Category | |
|---|---|***
***
Hypocotyl length (mm)
Ws
R1
R2
Col0
OE_1
OE_2
G
RNAi-2
WT
RNAi-1
400
400
400
2c
4c
8c
16c
2c
4c
8c
16c
32c
2c
4c
8c
16c
32c
320
320
320
240
240
240
Counts
Counts
Counts
160
160
160
80
80
80
0
0
0
1
1
1
10
100
1000
10
100
1000
10
100
1000
FL1
FL1
FL1

## Slide 2
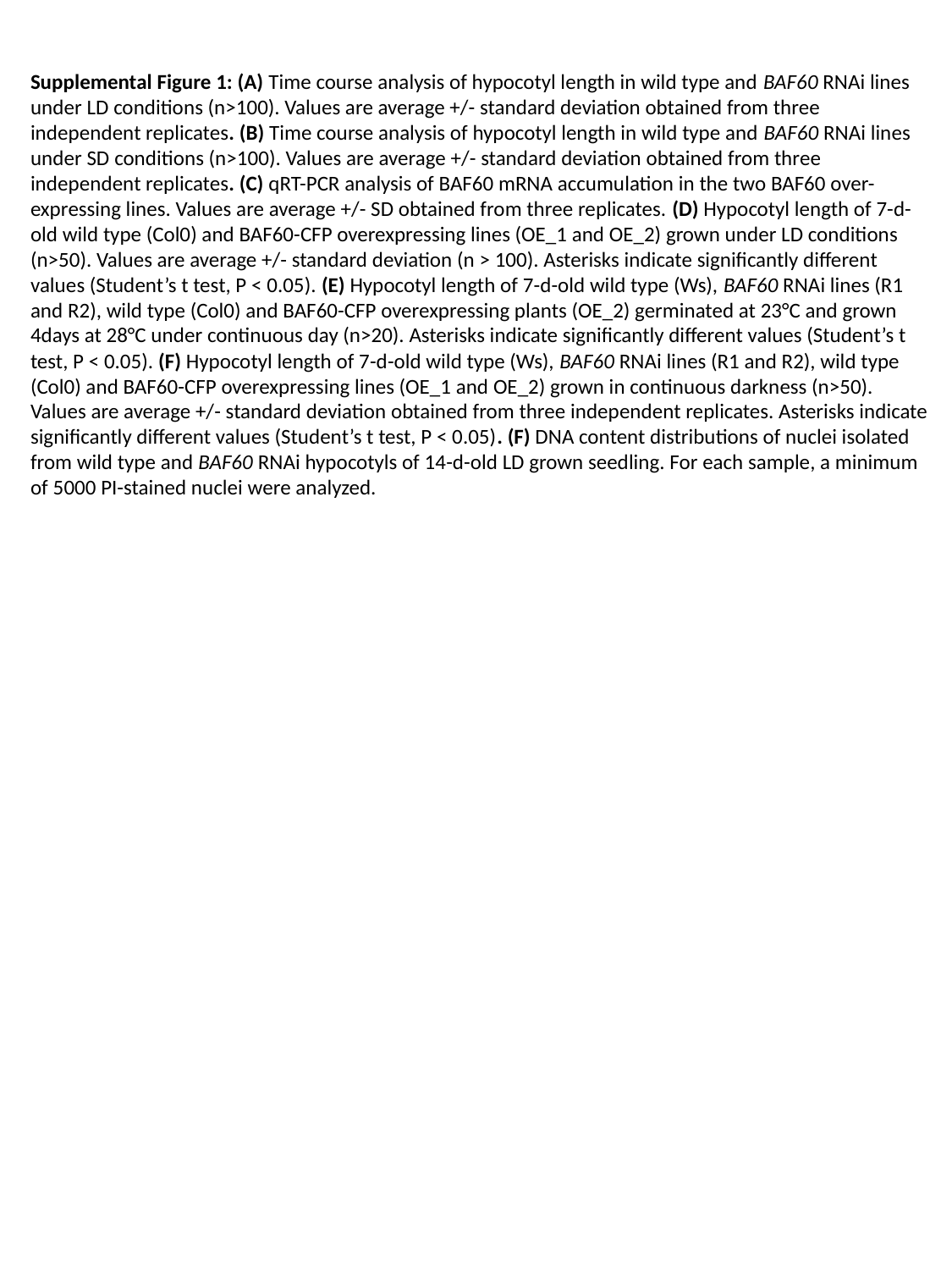

Supplemental Figure 1: (A) Time course analysis of hypocotyl length in wild type and BAF60 RNAi lines under LD conditions (n>100). Values are average +/- standard deviation obtained from three independent replicates. (B) Time course analysis of hypocotyl length in wild type and BAF60 RNAi lines under SD conditions (n>100). Values are average +/- standard deviation obtained from three independent replicates. (C) qRT-PCR analysis of BAF60 mRNA accumulation in the two BAF60 over-expressing lines. Values are average +/- SD obtained from three replicates. (D) Hypocotyl length of 7-d-old wild type (Col0) and BAF60-CFP overexpressing lines (OE_1 and OE_2) grown under LD conditions (n>50). Values are average +/- standard deviation (n > 100). Asterisks indicate significantly different values (Student’s t test, P < 0.05). (E) Hypocotyl length of 7-d-old wild type (Ws), BAF60 RNAi lines (R1 and R2), wild type (Col0) and BAF60-CFP overexpressing plants (OE_2) germinated at 23°C and grown 4days at 28°C under continuous day (n>20). Asterisks indicate significantly different values (Student’s t test, P < 0.05). (F) Hypocotyl length of 7-d-old wild type (Ws), BAF60 RNAi lines (R1 and R2), wild type (Col0) and BAF60-CFP overexpressing lines (OE_1 and OE_2) grown in continuous darkness (n>50). Values are average +/- standard deviation obtained from three independent replicates. Asterisks indicate significantly different values (Student’s t test, P < 0.05). (F) DNA content distributions of nuclei isolated from wild type and BAF60 RNAi hypocotyls of 14-d-old LD grown seedling. For each sample, a minimum of 5000 PI-stained nuclei were analyzed.

## Slide 3
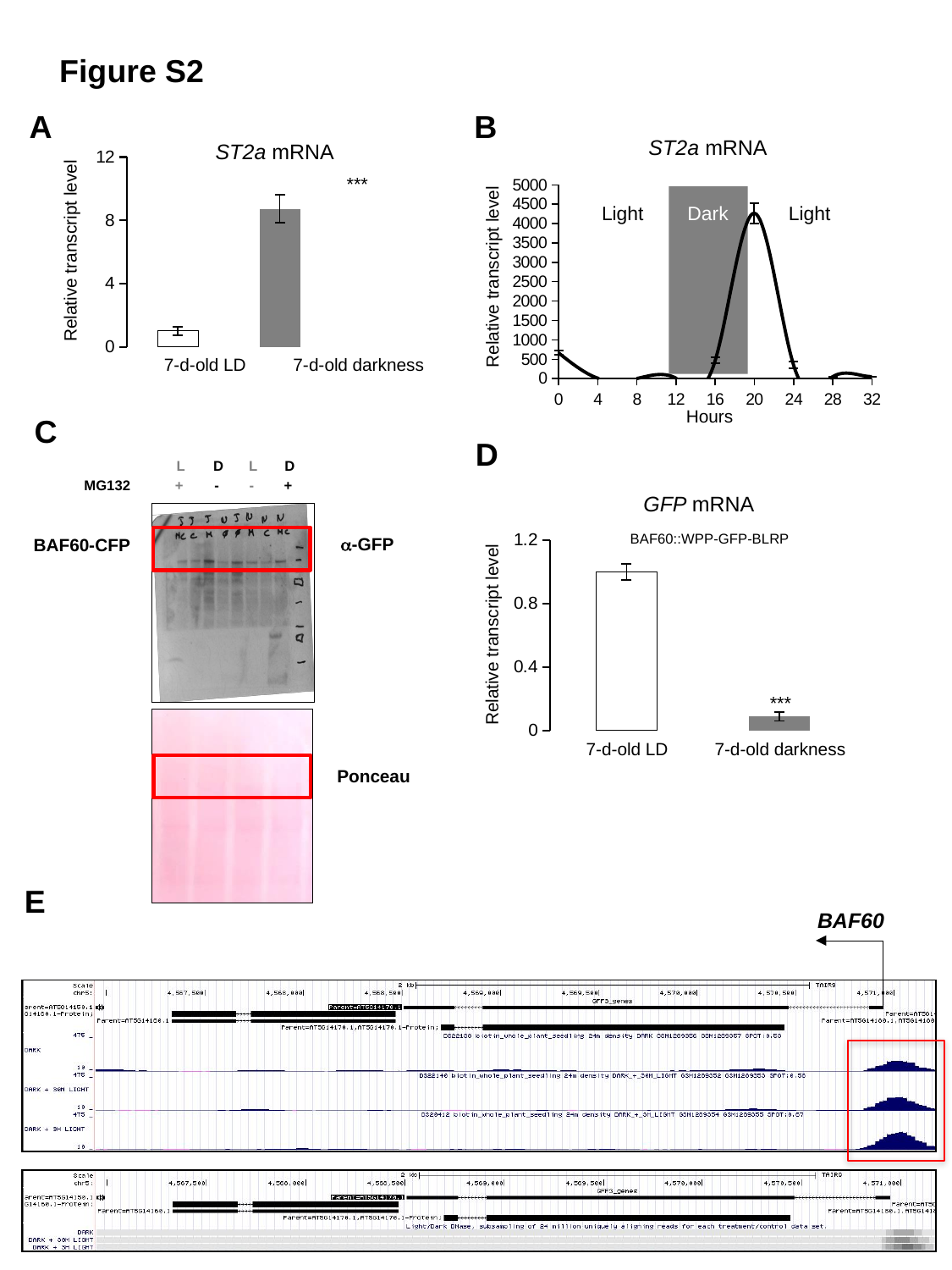

Figure S2
A
B
ST2a mRNA
ST2a mRNA
[unsupported chart]
***
### Chart
| Category | |
|---|---|
Light
Dark
Light
Relative transcript level
Relative transcript level
7-d-old LD
7-d-old darkness
Hours
C
D
L
D
L
D
MG132
+
-
-
+
GFP mRNA
BAF60::WPP-GFP-BLRP
a-GFP
BAF60-CFP
[unsupported chart]
Relative transcript level
***
7-d-old LD
7-d-old darkness
Ponceau
E
BAF60

## Slide 4
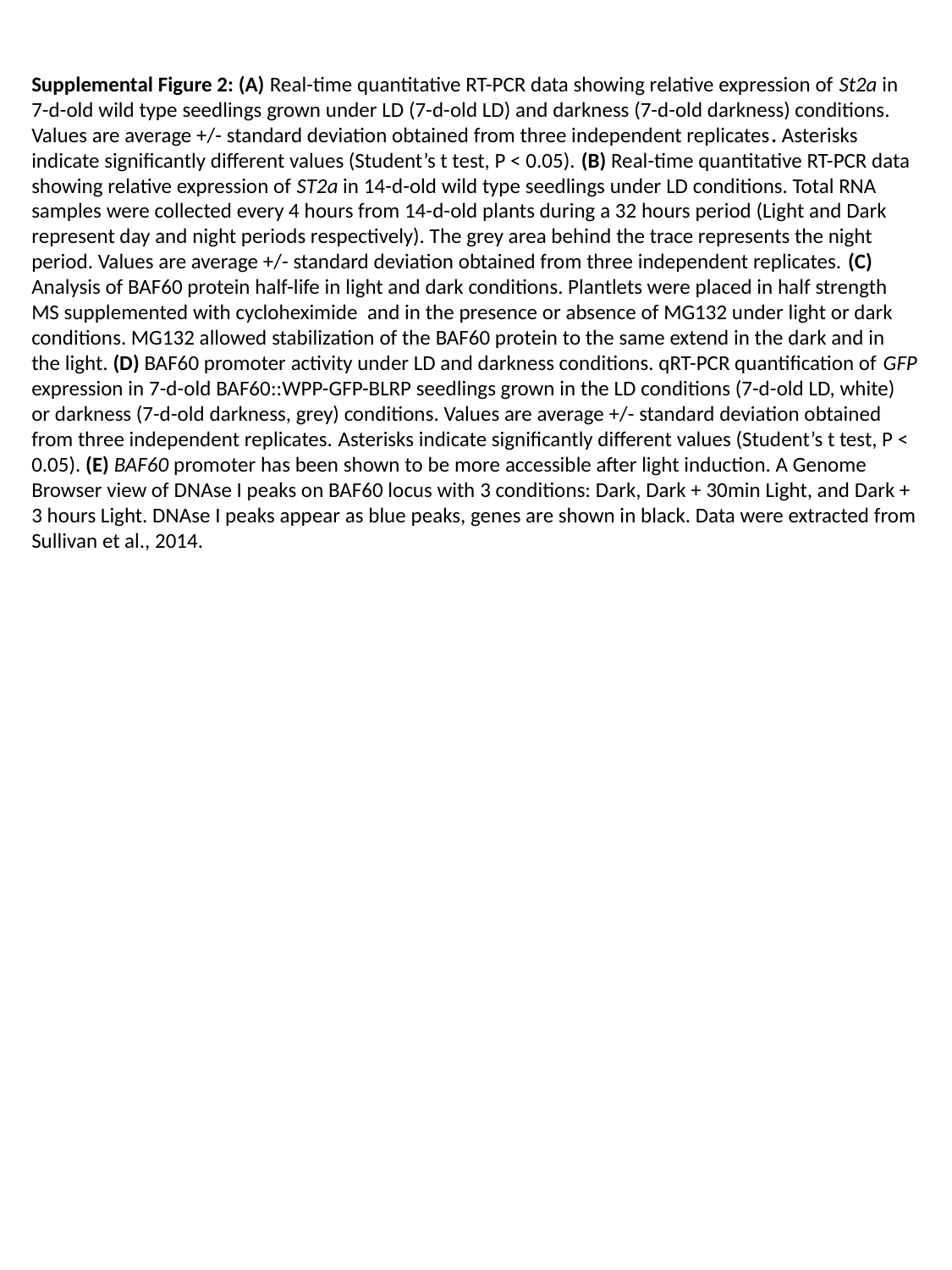

Supplemental Figure 2: (A) Real-time quantitative RT-PCR data showing relative expression of St2a in 7-d-old wild type seedlings grown under LD (7-d-old LD) and darkness (7-d-old darkness) conditions. Values are average +/- standard deviation obtained from three independent replicates. Asterisks indicate significantly different values (Student’s t test, P < 0.05). (B) Real-time quantitative RT-PCR data showing relative expression of ST2a in 14-d-old wild type seedlings under LD conditions. Total RNA samples were collected every 4 hours from 14-d-old plants during a 32 hours period (Light and Dark represent day and night periods respectively). The grey area behind the trace represents the night period. Values are average +/- standard deviation obtained from three independent replicates. (C) Analysis of BAF60 protein half-life in light and dark conditions. Plantlets were placed in half strength MS supplemented with cycloheximide and in the presence or absence of MG132 under light or dark conditions. MG132 allowed stabilization of the BAF60 protein to the same extend in the dark and in the light. (D) BAF60 promoter activity under LD and darkness conditions. qRT-PCR quantification of GFP expression in 7-d-old BAF60::WPP-GFP-BLRP seedlings grown in the LD conditions (7-d-old LD, white) or darkness (7-d-old darkness, grey) conditions. Values are average +/- standard deviation obtained from three independent replicates. Asterisks indicate significantly different values (Student’s t test, P < 0.05). (E) BAF60 promoter has been shown to be more accessible after light induction. A Genome Browser view of DNAse I peaks on BAF60 locus with 3 conditions: Dark, Dark + 30min Light, and Dark + 3 hours Light. DNAse I peaks appear as blue peaks, genes are shown in black. Data were extracted from Sullivan et al., 2014.

## Slide 5
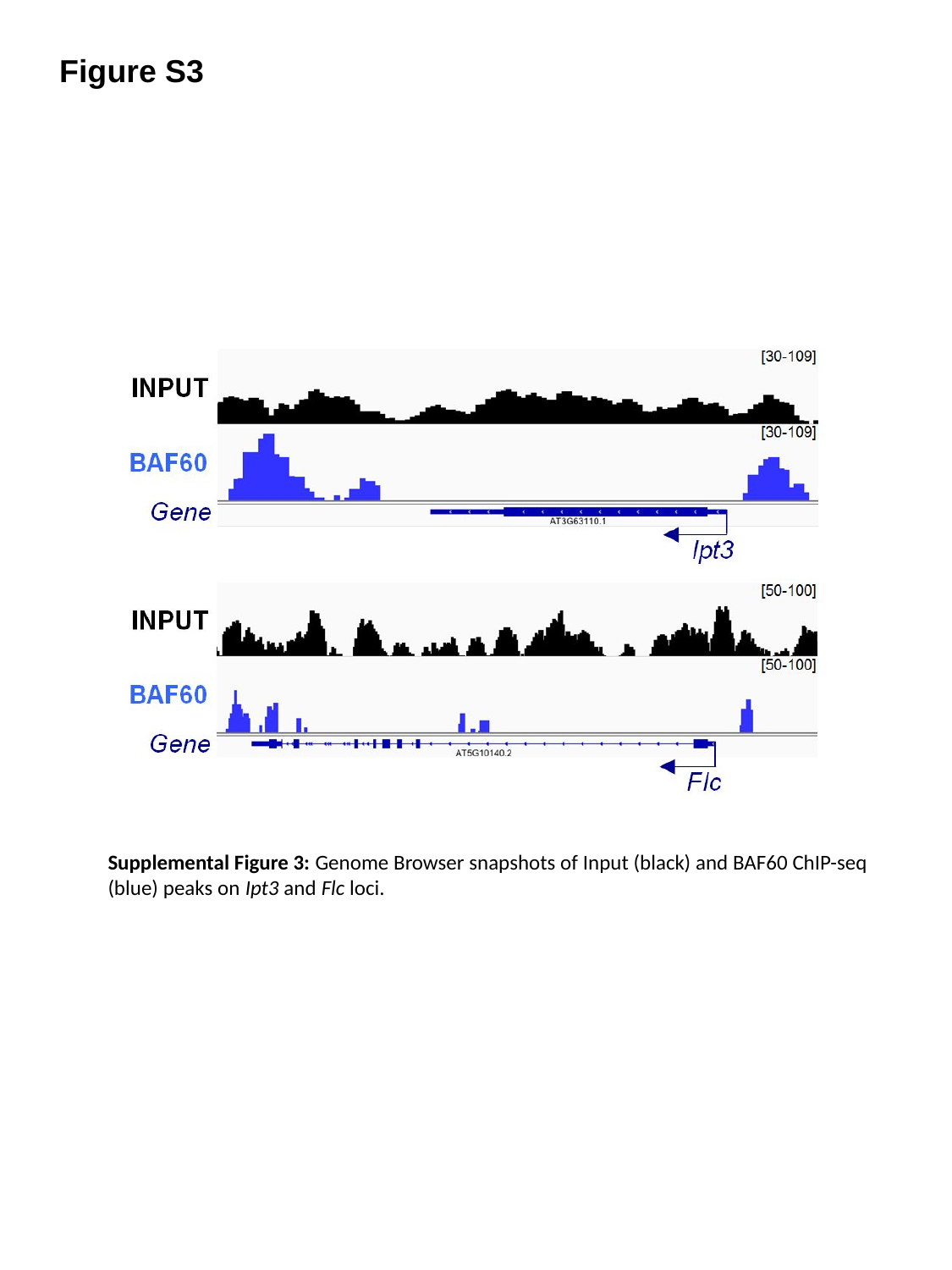

Figure S3
Supplemental Figure 3: Genome Browser snapshots of Input (black) and BAF60 ChIP-seq (blue) peaks on Ipt3 and Flc loci.

## Slide 6
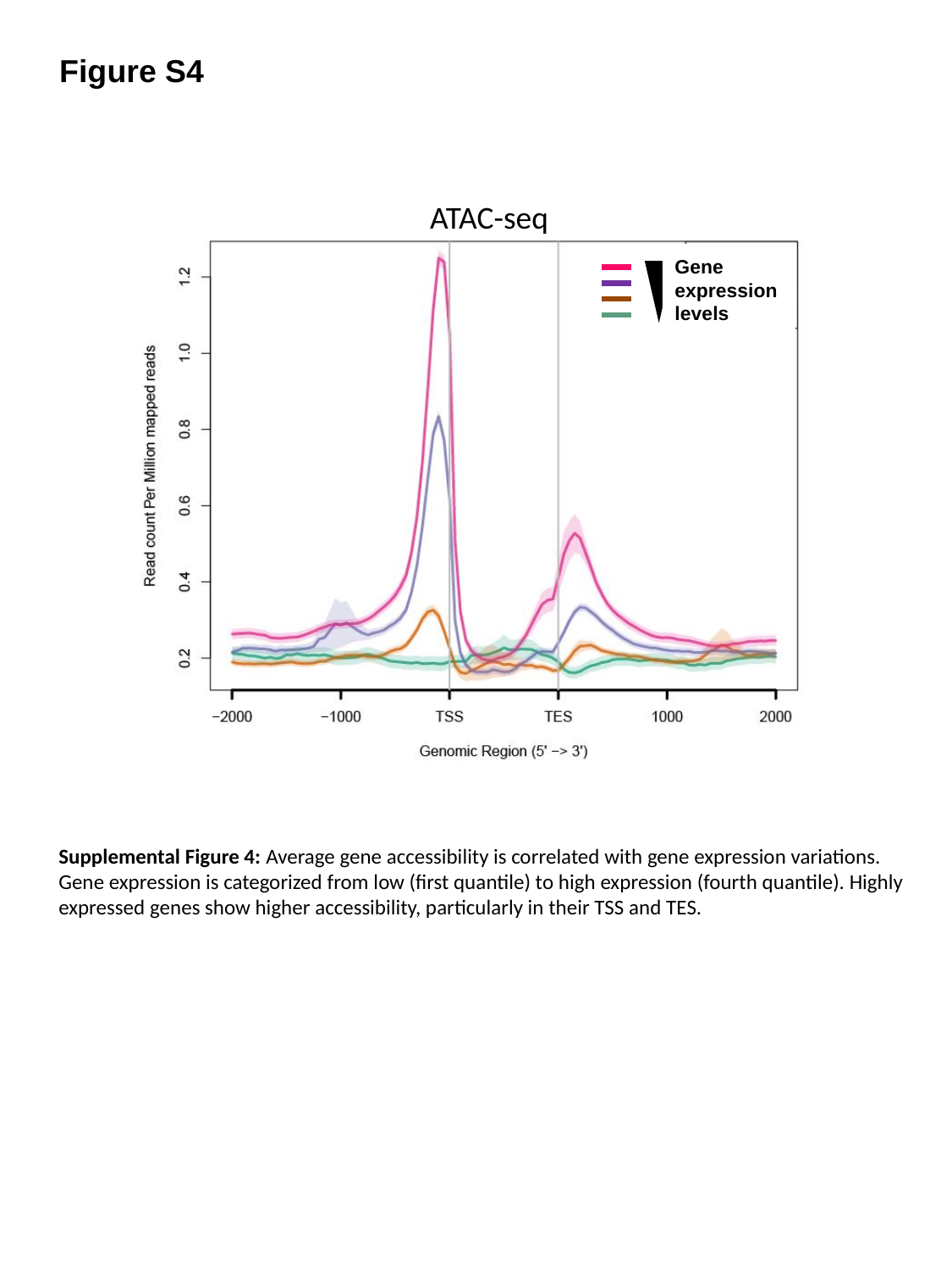

Figure S4
ATAC-seq
Gene expression levels
Supplemental Figure 4: Average gene accessibility is correlated with gene expression variations. Gene expression is categorized from low (first quantile) to high expression (fourth quantile). Highly expressed genes show higher accessibility, particularly in their TSS and TES.

## Slide 7
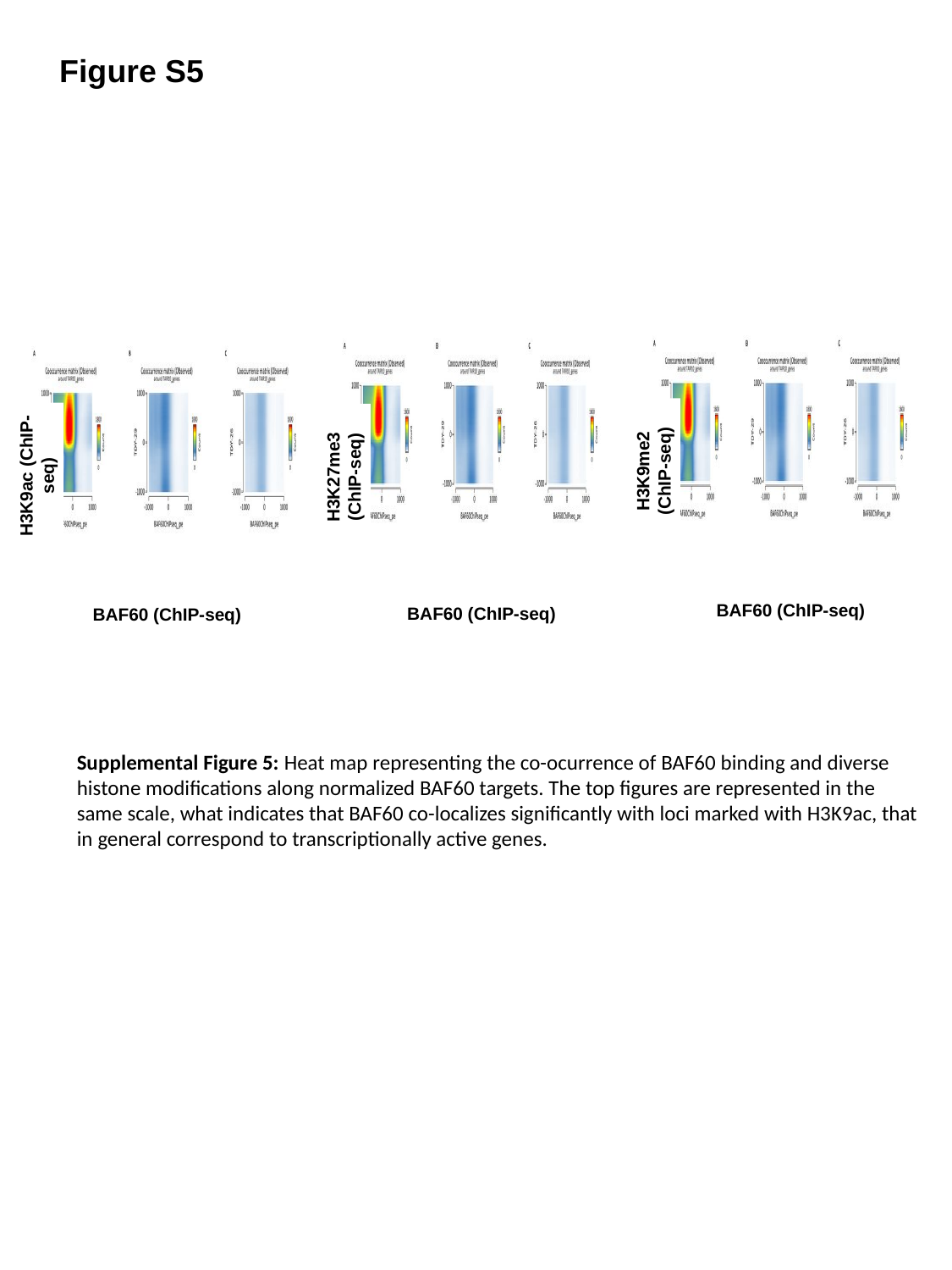

Figure S5
H3K9me2 (ChIP-seq)
H3K9ac (ChIP-seq)
H3K27me3 (ChIP-seq)
BAF60 (ChIP-seq)
BAF60 (ChIP-seq)
BAF60 (ChIP-seq)
Supplemental Figure 5: Heat map representing the co-ocurrence of BAF60 binding and diverse histone modifications along normalized BAF60 targets. The top figures are represented in the same scale, what indicates that BAF60 co-localizes significantly with loci marked with H3K9ac, that in general correspond to transcriptionally active genes.

## Slide 8
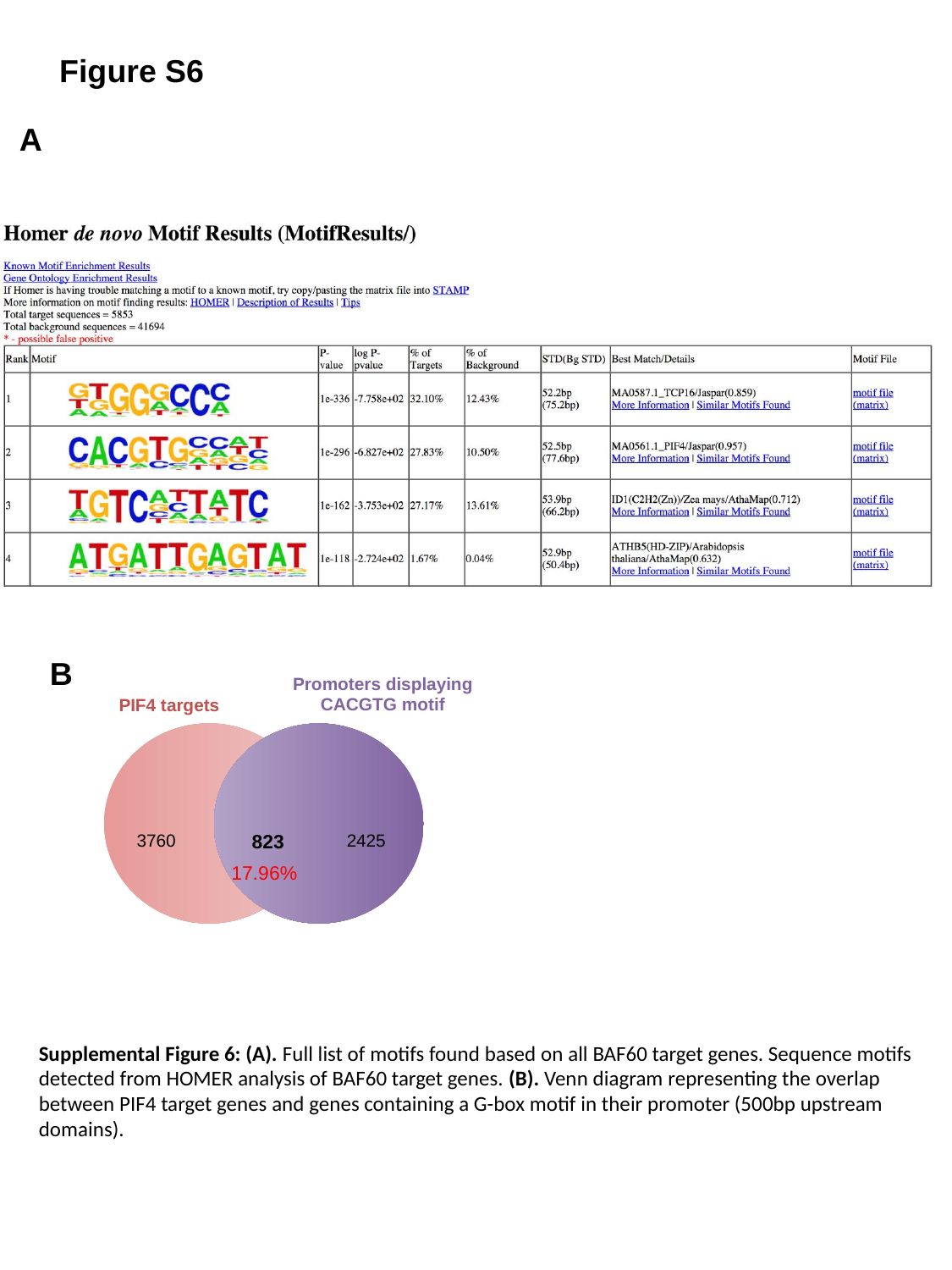

Figure S6
A
B
Promoters displaying
CACGTG motif
PIF4 targets
3760
823
2425
17.96%
Supplemental Figure 6: (A). Full list of motifs found based on all BAF60 target genes. Sequence motifs detected from HOMER analysis of BAF60 target genes. (B). Venn diagram representing the overlap between PIF4 target genes and genes containing a G-box motif in their promoter (500bp upstream domains).

## Slide 9
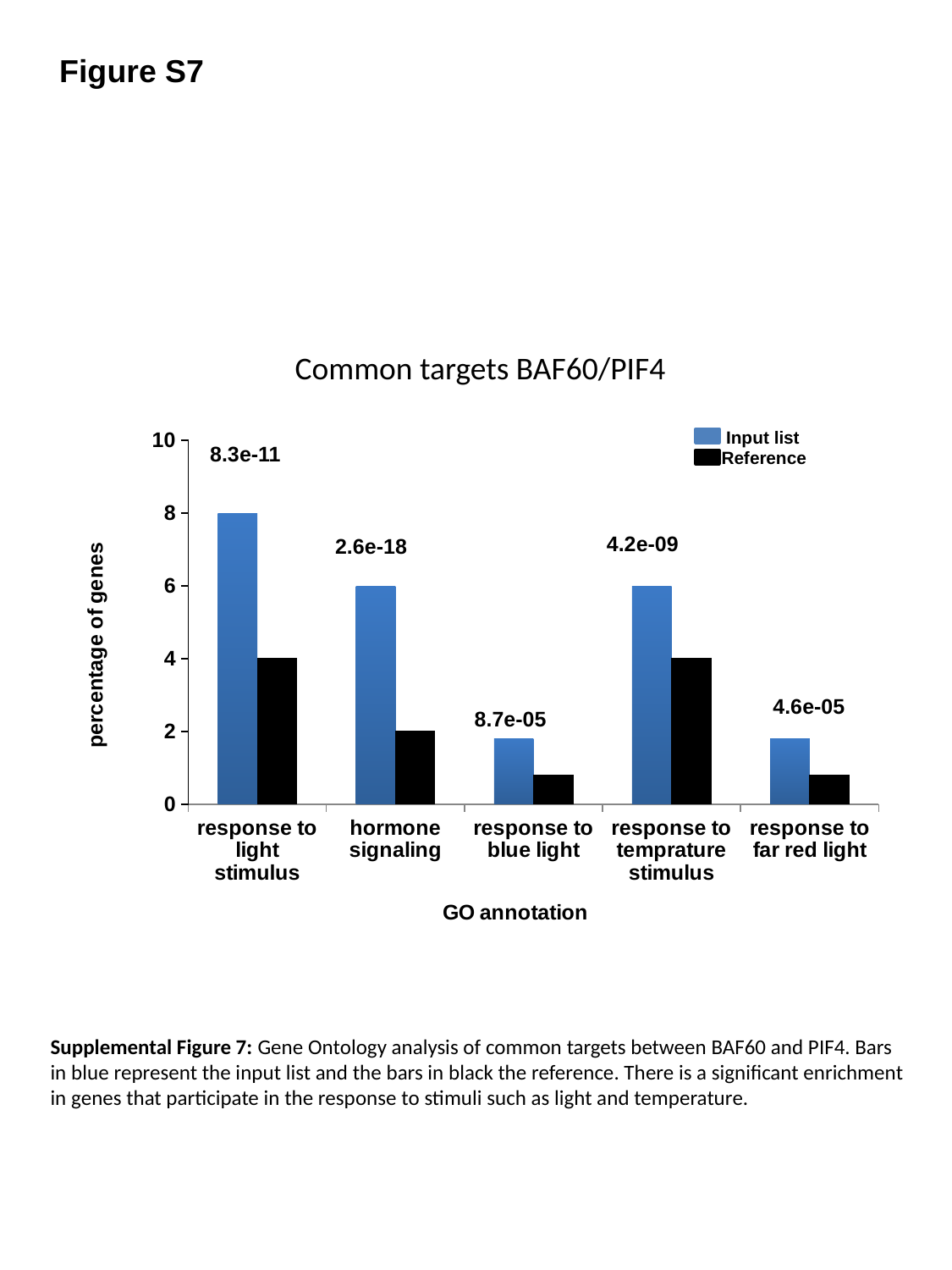

Figure S7
Common targets BAF60/PIF4
### Chart
| Category | Input list | Background/reference |
|---|---|---|
| response to light stimulus | 8.0 | 4.0 |
| hormone signaling | 6.0 | 2.0 |
| response to blue light | 1.8 | 0.8 |
| response to temprature stimulus | 6.0 | 4.0 |
| response to far red light | 1.8 | 0.8 |Input list
8.3e-11
Reference
4.2e-09
2.6e-18
4.6e-05
8.7e-05
Supplemental Figure 7: Gene Ontology analysis of common targets between BAF60 and PIF4. Bars in blue represent the input list and the bars in black the reference. There is a significant enrichment in genes that participate in the response to stimuli such as light and temperature.

## Slide 10
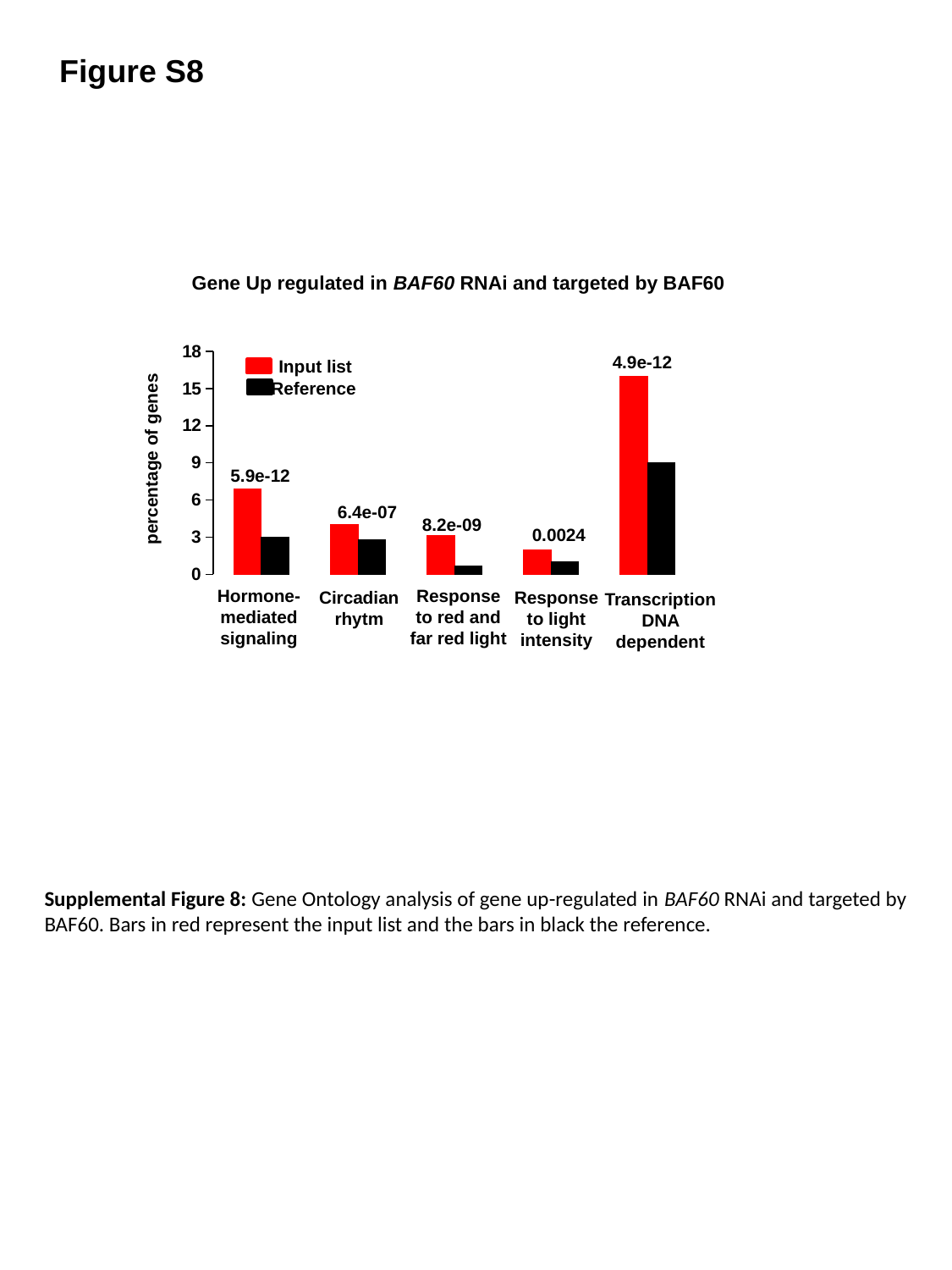

Figure S8
Gene Up regulated in BAF60 RNAi and targeted by BAF60
### Chart
| Category | Input list | Background/reference |
|---|---|---|
| hormone-mediated signaling | 6.9 | 3.0 |
| response to red and far red light | 4.0 | 2.8 |
| circadian rhythm | 3.1 | 0.7 |
| response to light intensity | 2.0 | 1.0 |
| transcription, DNA-dependent | 16.0 | 9.0 |4.9e-12
Input list
Reference
5.9e-12
6.4e-07
8.2e-09
0.0024
percentage of genes
Hormone-mediated signaling
Response to red and far red light
Circadian
rhytm
Response to light intensity
Transcription DNA dependent
Supplemental Figure 8: Gene Ontology analysis of gene up-regulated in BAF60 RNAi and targeted by BAF60. Bars in red represent the input list and the bars in black the reference.

## Slide 11
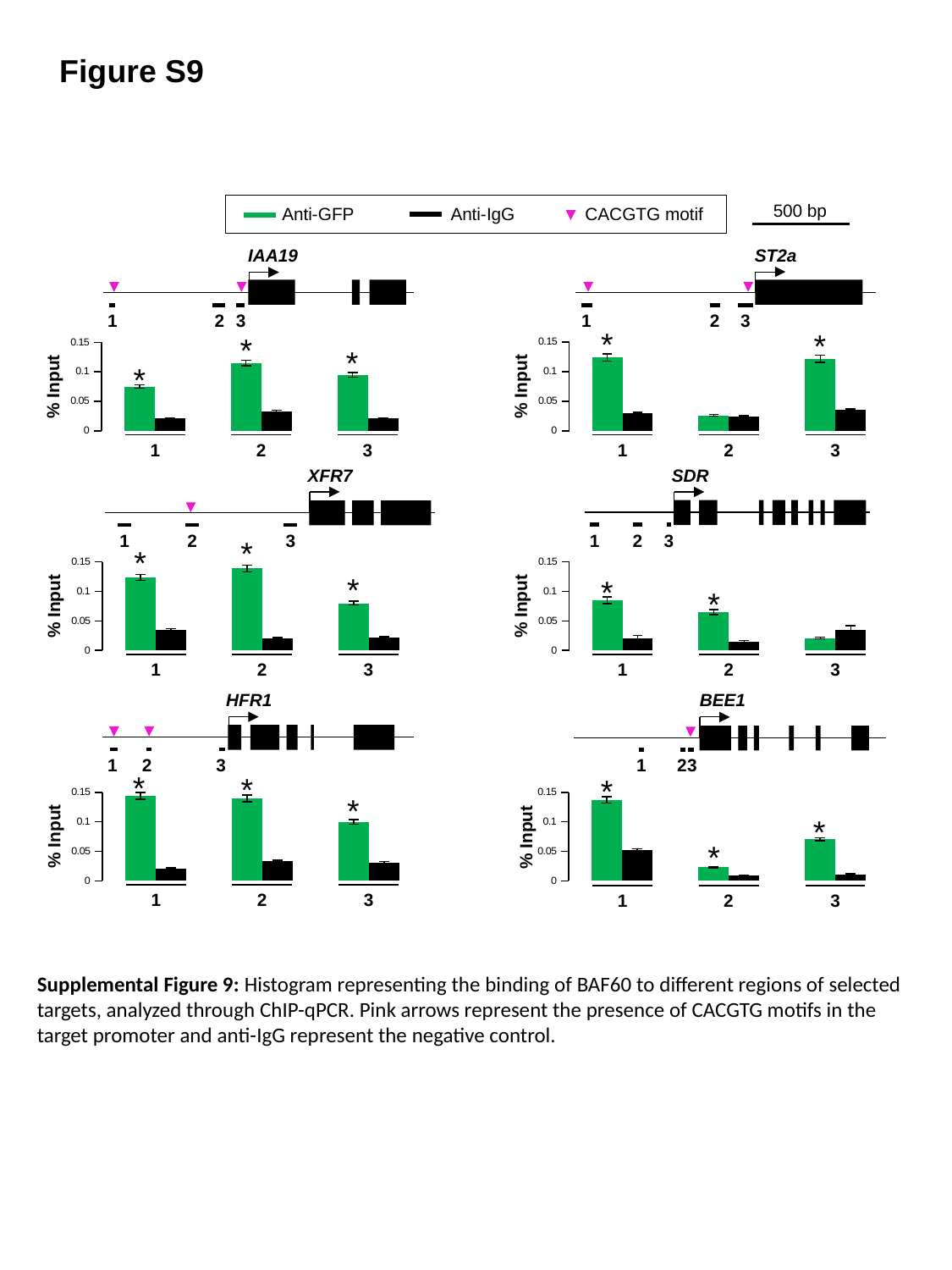

Figure S9
500 bp
Anti-GFP
Anti-IgG
CACGTG motif
ST2a
IAA19
1
2
3
2
3
1
*
*
*
### Chart
| Category | 0,124 0,026 0,122 | |
|---|---|---|
| | 0.124 | 0.03 |
| | 0.026 | 0.024 |
| | 0.122 | 0.035 |
### Chart
| Category | | |
|---|---|---|
| | 0.075 | 0.021 |
| | 0.115 | 0.033 |
| | 0.095 | 0.021 |*
*
% Input
% Input
1
2
3
1
2
3
XFR7
SDR
1
2
3
1
2
3
*
*
### Chart
| Category | | |
|---|---|---|
| | 0.124 | 0.035 |
| | 0.139 | 0.021 |
| | 0.08 | 0.022 |
### Chart
| Category | | |
|---|---|---|
| | 0.085 | 0.021 |
| | 0.065 | 0.014 |
| | 0.021 | 0.035 |*
*
*
% Input
% Input
1
2
3
1
2
3
HFR1
BEE1
1
2
3
1
2
3
*
*
*
*
### Chart
| Category | | |
|---|---|---|
| | 0.144 | 0.021 |
| | 0.14 | 0.033 |
| | 0.1 | 0.031 |
### Chart
| Category | | |
|---|---|---|
| IgG | 0.137288279694154 | 0.0516352091524169 |*
% Input
% Input
*
1
2
3
1
2
3
Supplemental Figure 9: Histogram representing the binding of BAF60 to different regions of selected targets, analyzed through ChIP-qPCR. Pink arrows represent the presence of CACGTG motifs in the target promoter and anti-IgG represent the negative control.

## Slide 12
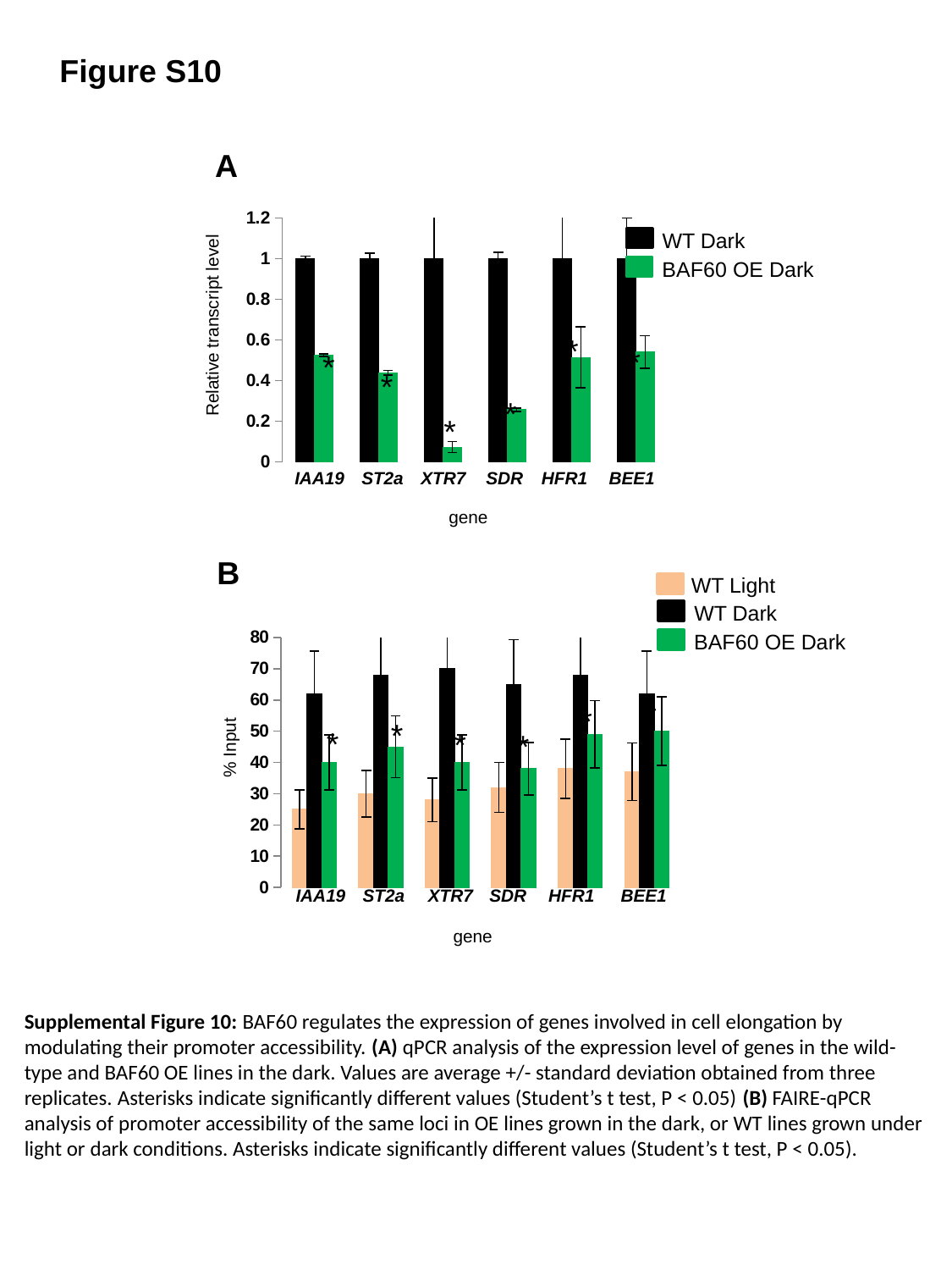

Figure S10
A
### Chart
| Category | | |
|---|---|---|WT Dark
BAF60 OE Dark
Relative transcript level
*
*
*
*
*
*
IAA19
ST2a
XTR7
SDR
HFR1
 BEE1
gene
B
WT Light
WT Dark
### Chart
| Category | | | |
|---|---|---|---|BAF60 OE Dark
*
*
*
*
*
*
% Input
IAA19
ST2a
XTR7
SDR
HFR1
 BEE1
gene
Supplemental Figure 10: BAF60 regulates the expression of genes involved in cell elongation by modulating their promoter accessibility. (A) qPCR analysis of the expression level of genes in the wild-type and BAF60 OE lines in the dark. Values are average +/- standard deviation obtained from three replicates. Asterisks indicate significantly different values (Student’s t test, P < 0.05) (B) FAIRE-qPCR analysis of promoter accessibility of the same loci in OE lines grown in the dark, or WT lines grown under light or dark conditions. Asterisks indicate significantly different values (Student’s t test, P < 0.05).

## Slide 13
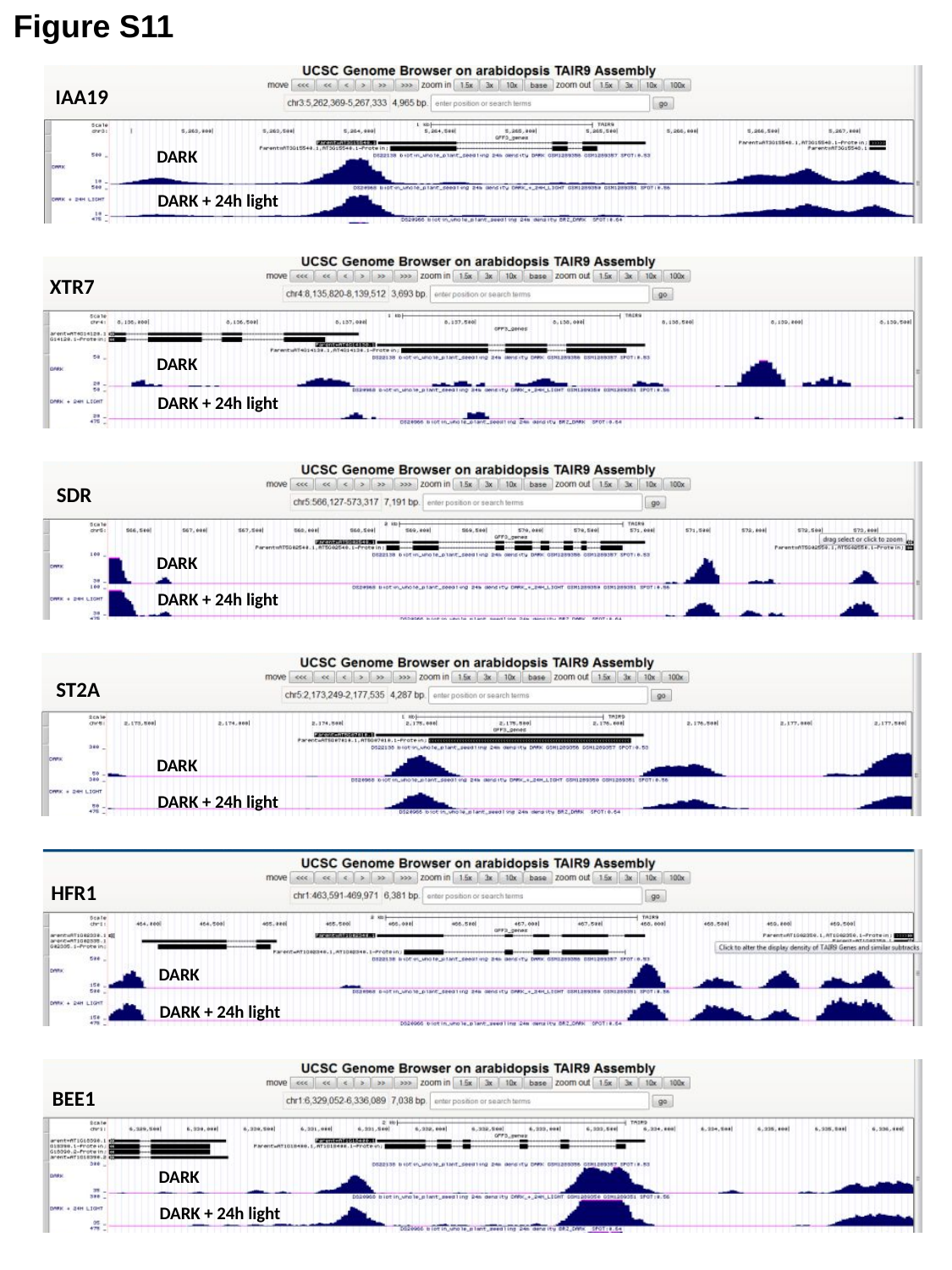

Figure S11
IAA19
DARK
DARK + 24h light
XTR7
DARK
DARK + 24h light
SDR
DARK
DARK + 24h light
ST2A
DARK
DARK + 24h light
HFR1
DARK
DARK + 24h light
BEE1
DARK
DARK + 24h light

## Slide 14
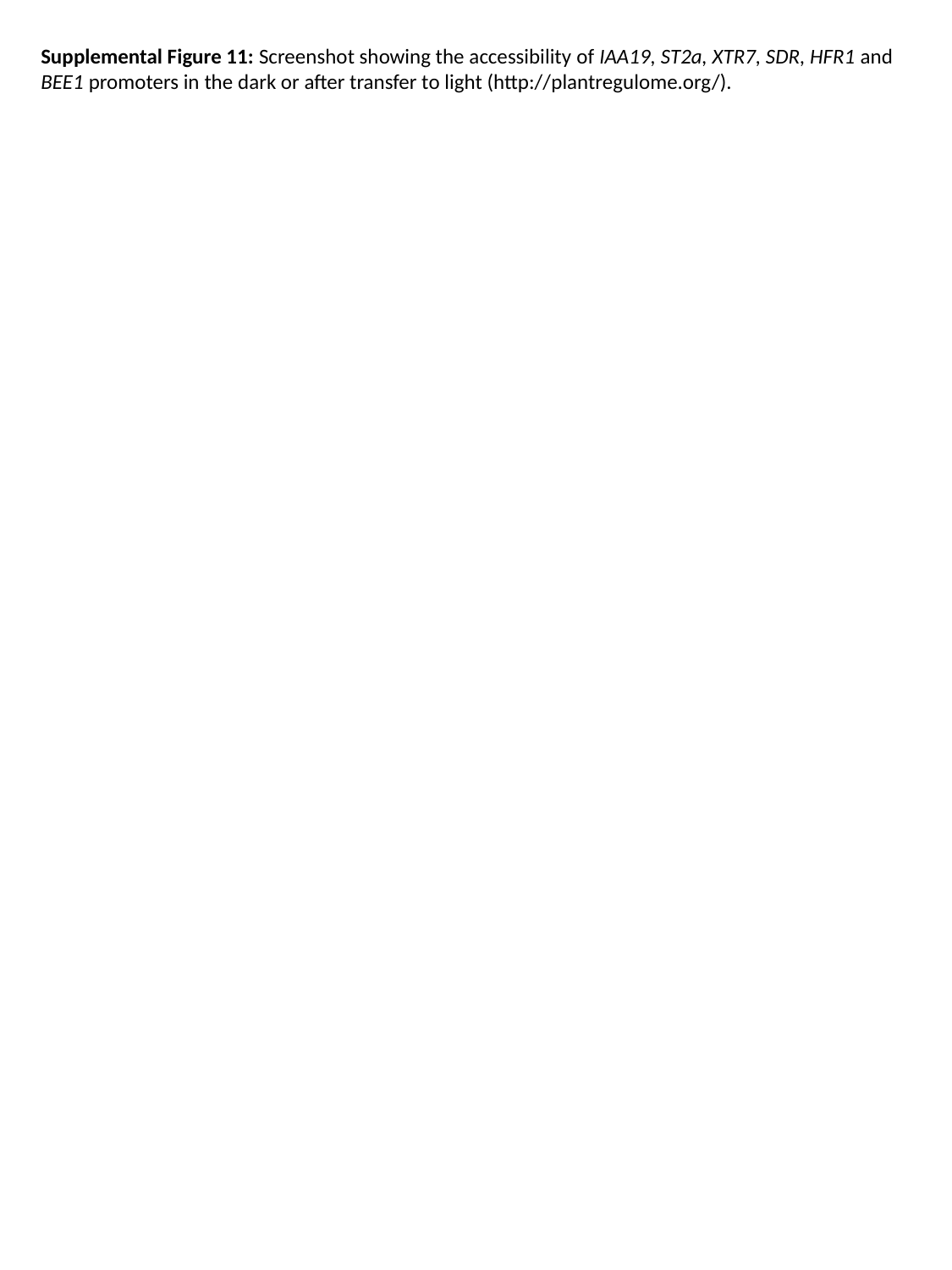

Supplemental Figure 11: Screenshot showing the accessibility of IAA19, ST2a, XTR7, SDR, HFR1 and BEE1 promoters in the dark or after transfer to light (http://plantregulome.org/).

## Slide 15
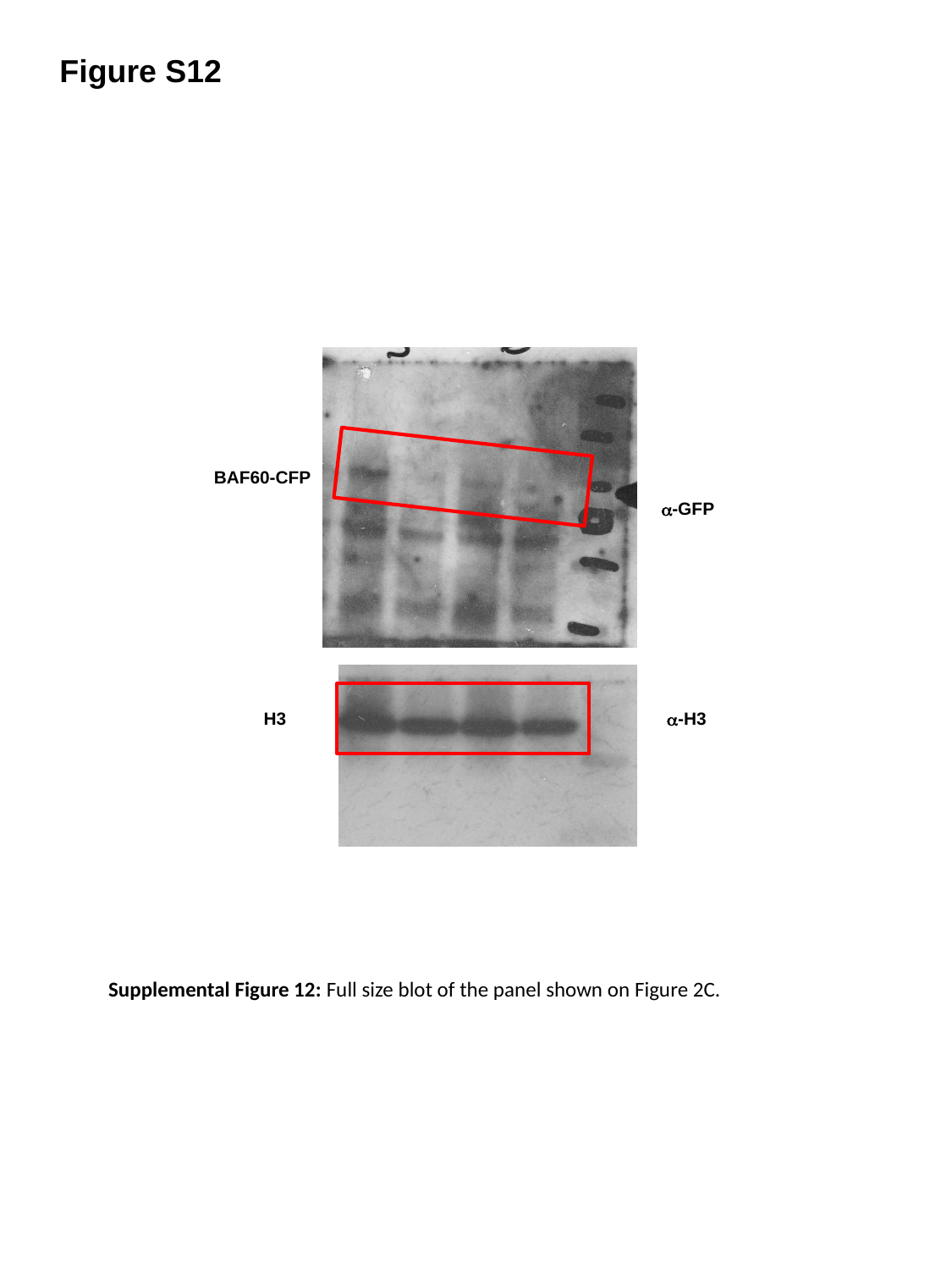

Figure S12
BAF60-CFP
a-GFP
a-H3
H3
Supplemental Figure 12: Full size blot of the panel shown on Figure 2C.

## Slide 16
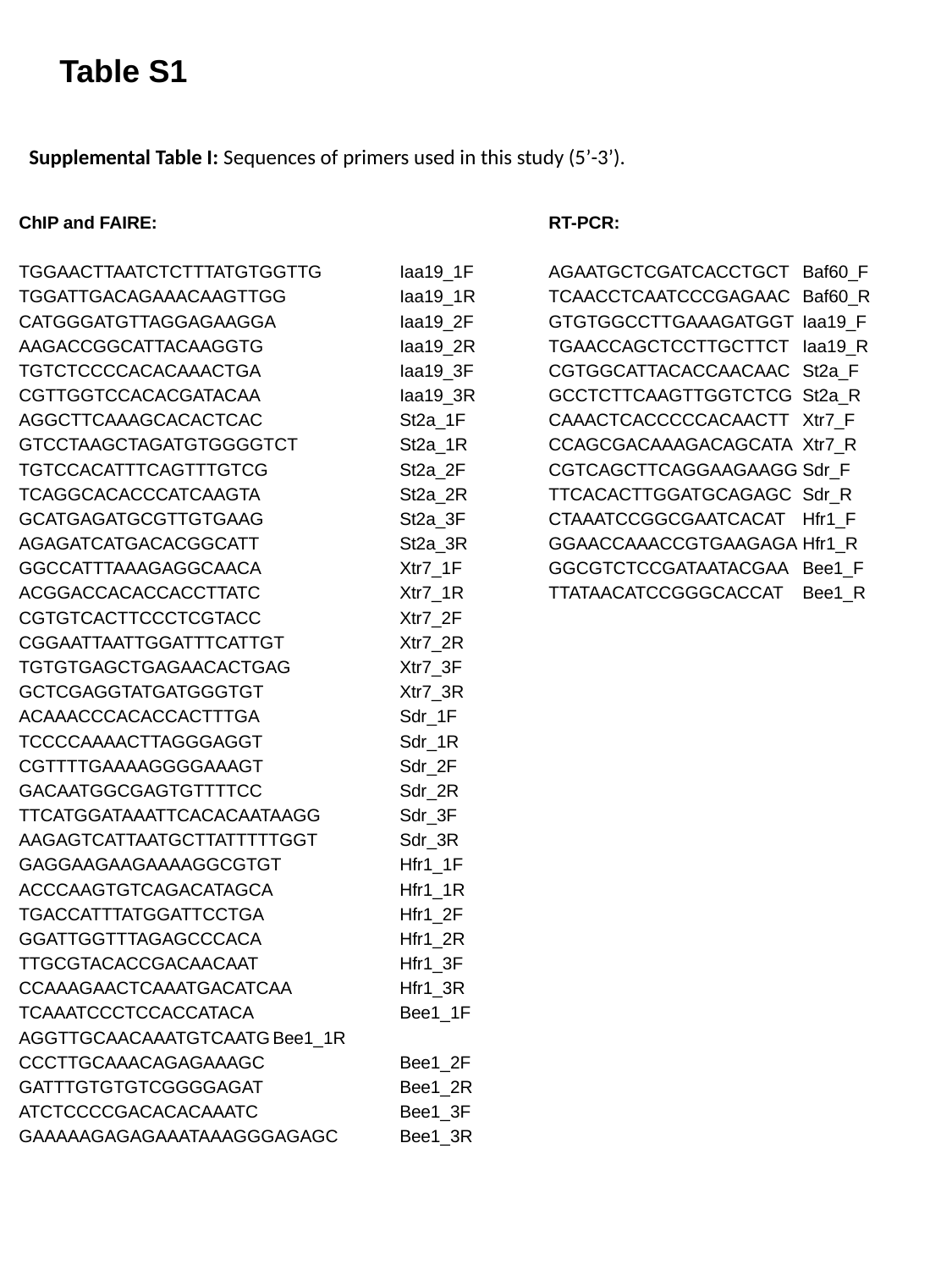

Table S1
Supplemental Table I: Sequences of primers used in this study (5’-3’).
ChIP and FAIRE:
TGGAACTTAATCTCTTTATGTGGTTG	Iaa19_1F
TGGATTGACAGAAACAAGTTGG	Iaa19_1R
CATGGGATGTTAGGAGAAGGA	Iaa19_2F
AAGACCGGCATTACAAGGTG		Iaa19_2R
TGTCTCCCCACACAAACTGA		Iaa19_3F
CGTTGGTCCACACGATACAA		Iaa19_3R
AGGCTTCAAAGCACACTCAC		St2a_1F
GTCCTAAGCTAGATGTGGGGTCT	St2a_1R
TGTCCACATTTCAGTTTGTCG		St2a_2F
TCAGGCACACCCATCAAGTA		St2a_2R
GCATGAGATGCGTTGTGAAG		St2a_3F
AGAGATCATGACACGGCATT		St2a_3R
GGCCATTTAAAGAGGCAACA		Xtr7_1F
ACGGACCACACCACCTTATC		Xtr7_1R
CGTGTCACTTCCCTCGTACC		Xtr7_2F
CGGAATTAATTGGATTTCATTGT	Xtr7_2R
TGTGTGAGCTGAGAACACTGAG	Xtr7_3F
GCTCGAGGTATGATGGGTGT		Xtr7_3R
ACAAACCCACACCACTTTGA		Sdr_1F
TCCCCAAAACTTAGGGAGGT		Sdr_1R
CGTTTTGAAAAGGGGAAAGT		Sdr_2F
GACAATGGCGAGTGTTTTCC		Sdr_2R
TTCATGGATAAATTCACACAATAAGG	Sdr_3F
AAGAGTCATTAATGCTTATTTTTGGT	Sdr_3R
GAGGAAGAAGAAAAGGCGTGT	Hfr1_1F
ACCCAAGTGTCAGACATAGCA	Hfr1_1R
TGACCATTTATGGATTCCTGA		Hfr1_2F
GGATTGGTTTAGAGCCCACA		Hfr1_2R
TTGCGTACACCGACAACAAT		Hfr1_3F
CCAAAGAACTCAAATGACATCAA	Hfr1_3R
TCAAATCCCTCCACCATACA		Bee1_1F
AGGTTGCAACAAATGTCAATG	Bee1_1R
CCCTTGCAAACAGAGAAAGC		Bee1_2F
GATTTGTGTGTCGGGGAGAT		Bee1_2R
ATCTCCCCGACACACAAATC		Bee1_3F
GAAAAAGAGAGAAATAAAGGGAGAGC	Bee1_3R
RT-PCR:
AGAATGCTCGATCACCTGCT	Baf60_F
TCAACCTCAATCCCGAGAAC	Baf60_R
GTGTGGCCTTGAAAGATGGT	Iaa19_F
TGAACCAGCTCCTTGCTTCT	Iaa19_R
CGTGGCATTACACCAACAAC	St2a_F
GCCTCTTCAAGTTGGTCTCG	St2a_R
CAAACTCACCCCCACAACTT	Xtr7_F
CCAGCGACAAAGACAGCATA	Xtr7_R
CGTCAGCTTCAGGAAGAAGG	Sdr_F
TTCACACTTGGATGCAGAGC	Sdr_R
CTAAATCCGGCGAATCACAT	Hfr1_F
GGAACCAAACCGTGAAGAGA	Hfr1_R
GGCGTCTCCGATAATACGAA	Bee1_F
TTATAACATCCGGGCACCAT	Bee1_R
